# Consistent prokaryotic community patterns along the radial root axis of two *Zea mays* L. landraces across two distinct field locations

**DOI:** 10.3389/fmicb.2024.1386476

**Published:** 2024-07-17

**Authors:** Nicolas Tyborski, Tina Koehler, Franziska A. Steiner, Shu-Yin Tung, Andreas J. Wild, Andrea Carminati, Carsten W. Mueller, Alix Vidal, Sebastian Wolfrum, Johanna Pausch, Tillmann Lueders

**Affiliations:** ^1^Ecological Microbiology, Bayreuth Center of Ecology and Environmental Research (BayCEER), University of Bayreuth, Bayreuth, Germany; ^2^Root-Soil Interaction, TUM School of Life Sciences, Technical University of Munich, Freising, Germany; ^3^Soil Science, TUM School of Life Sciences, Technical University of Munich, Freising, Germany; ^4^Institute for Agroecology and Organic Farming, Bavarian State Research Center for Agriculture (LfL), Freising, Germany; ^5^TUM School of Life Sciences, Technical University of Munich, Freising, Germany; ^6^Agroecology, Bayreuth Center of Ecology and Environmental Research (BayCEER), University of Bayreuth, Bayreuth, Germany; ^7^Physics of Soils and Terrestrial Ecosystems, Department of Environmental Systems Science, ETH Zurich, Zurich, Switzerland; ^8^Soil Science, Institute of Ecology, Technical University of Berlin, Berlin, Germany; ^9^Department of Geosciences and Natural Resource Management, University of Copenhagen, Copenhagen, Denmark; ^10^Soil Biology, Wageningen University and Research, Wageningen, Netherlands

**Keywords:** microbiome, root endosphere, rhizosphere, differential abundance analysis, maize (*Zea mays* L.), landraces, ammonia-oxidizers

## Abstract

The close interconnection of plants with rhizosphere- and root-associated microorganisms is well recognized, and high expectations are raised for considering their symbioses in the breeding of future crop varieties. However, it is unclear how consistently plant-mediated selection, a potential target in crop breeding, influences microbiome members compared to selection imposed by the agricultural environment. Landraces may have traits shaping their microbiome, which were lost during the breeding of modern varieties, but knowledge about this is scarce. We investigated prokaryotic community composition along the radial root axis of two European maize (*Zea mays* L.) landraces. A sampling gradient included bulk soil, a distal and proximal rhizosphere fraction, and the root compartment. Our study was replicated at two field locations with differing edaphic and climatic conditions. Further, we tested for differences between two plant developmental stages and two precipitation treatments. Community data were generated by metabarcoding of the V4 SSU rRNA region. While communities were generally distinct between field sites, the effects of landrace variety, developmental stage, and precipitation treatment were comparatively weak and not statistically significant. Under all conditions, patterns in community composition corresponded strongly to the distance to the root. Changes in α- and β-diversity, as well as abundance shifts of many taxa along this gradient, were similar for both landraces and field locations. Most affected taxa belonged to a core microbiome present in all investigated samples. Remarkably, we observed consistent enrichment of *Actinobacteriota* (particularly *Streptomyces*, *Lechevalieria*) and *Pseudomonadota* (particularly *Sphingobium*) toward the root. Further, we report a depletion of ammonia-oxidizers along this axis at both field sites. We identified clear enrichment and depletion patterns in microbiome composition along the radial root axis of *Z. mays*. Many of these were consistent across two distinct field locations, plant developmental stages, precipitation treatments, and for both landraces. This suggests a considerable influence of plant-mediated effects on the microbiome. We propose that the affected taxa have key roles in the rhizosphere and root microbiome of *Z. mays*. Understanding the functions of these taxa appears highly relevant for the development of methods aiming to promote microbiome services for crops.

## 1 Introduction

The composition of rhizosphere and root-associated microbiomes has substantial influence on plant fitness and crop performance (Berendsen et al., 2012; Vandenkoornhuyse et al., 2015; Müller et al., 2016; Bai et al., 2022). However, although fostering plant traits that support the establishment of a microbiome with beneficial functions is suggested as a means to enhance desirable crop properties, this has remained largely unexploited in the breeding of crop varieties to date (Bakker et al., 2012; Pantigoso et al., 2022). One reason for this is likely the lack of knowledge about how predictably and effectively plant traits can influence microbial community composition in the rhizosphere and roots compared to the impact of location-specific environmental factors. Large-scale field experiments showed field location to be among the strongest factors explaining variation in the rhizosphere microbiome of modern inbred maize (*Zea mays* L.) (Peiffer et al., 2013; Walters et al., 2018). This demonstrates the predominant influence of edaphic properties and climatic conditions on the composition of rhizosphere microbiomes. Further, colonization of the rhizosphere and root occurs mainly from the surrounding soil (Fitzpatrick et al., 2020; Rüger et al., 2021). The microbial taxa present at a geographic location thus act as a seed bank of diversity that can become part of the rhizosphere and root-associated microbial community (Bulgarelli et al., 2013; Philippot et al., 2013).

From a plant perspective, it is advantageous to influence community composition in the rhizosphere and root in a way that fosters taxa with beneficial functions and suppresses detrimental taxa. Plants can achieve this through active modulation of rhizodeposition, e.g., the release of nutrients, exudates, border cells, and mucilage (Chaparro et al., 2013; Philippot et al., 2013; Kawasaki et al., 2016; Lareen et al., 2016). At the rhizoplane (the root surface) and in the endosphere (the internal space of roots), the microbiome is additionally shaped by the plant immune system (Bulgarelli et al., 2013). Consequently, the influence of environmental factors associated with the field location decreases with increasing proximity to the root. Resolving differences between the compartments bulk soil, rhizosphere, rhizoplane, and endosphere, a decrease in the influence of the field location with proximity to the root has been reported for rice (*Oryza sativa* L.) (Edwards et al., 2015, 2018) and modern hybrid varieties of *Z. mays* (Xiong et al., 2021). Differences in community composition between these compartments were generally larger than differences between field locations in these studies, highlighting the strong selective pressure imposed by the plant (Bulgarelli et al., 2013). The observation of plant species- or even variety-specific microbial communities (Walters et al., 2018; Xiong et al., 2021) provides further evidence for the considerable influence of plant-mediated factors on community composition in the rhizosphere and roots. Taxonomic richness usually decreases toward the root, indicating selection and specialization of the community (Edwards et al., 2015; Bai et al., 2023). This typically comes with increased dominance of members of the *Pseudomonadota*, *Actinomycetota*, *Bacteroidota*, and *Bacillota* compared to the surrounding soil (Xiong et al., 2021; Gardner et al., 2022; Bai et al., 2023). Further, stochastic processes such as random undirected processes, including priority effects, drift, and dispersal, are significant determinants of community composition (Maignien et al., 2014; Shi et al., 2018; Bonkowski et al., 2021; Zhong et al., 2022). However, it remains elusive how location-specific environmental factors, plant-mediated factors, and stochastic processes compare in their effect on the abundance of specific rhizosphere and root microbiome members.

To address this, we conducted a field experiment with *Z. mays*, replicated at two distinct field locations in southern Germany, differing in edaphic and climatic conditions. Additionally, we implemented a precipitation-reduced treatment to simulate further climatic differences and sampled at two times to assess the potential influence of the plant developmental stage on microbiome composition. The comparability of studies on the rhizosphere is often hampered by inconsistencies in the soil volume sampled since the choice of a sampling method is commonly guided by considerations about the amount of soil required for specific methods (Vetterlein et al., 2021). Additionally, a finer spatial resolution along the radial root axis has been emphasized as important for a holistic understanding of rhizosphere functions (Vetterlein et al., 2020). Therefore, we included a distal and proximal rhizosphere compartment in addition to bulk soil and root samples.

*Z. mays* is an important model plant (Strable and Scanlon, 2009) and one of the most economically relevant staple crops globally (Erenstein et al., 2022). Open-pollinated landraces, genetically heterogeneous varieties that are adapted to regional environmental conditions (Casañas et al., 2017), are gaining attention as a potential source of genetic diversity that could be used in future breeding efforts (Wissuwa et al., 2009; Böhm et al., 2017; Mayer et al., 2017). However, the vast majority of studies on the microbiome of *Z. mays* were conducted on modern, yield-optimized inbred lines or hybrid varieties. Landraces have been neglected in research on the microbiome of maize to date. To address this knowledge gap, we conducted our experiment with two European landraces of *Z. mays*, which were among the most popular maize varieties in central Europe before the 1950s and which were used as parental lines in the breeding of modern inbred varieties (Oettler et al., 1976; Barrière et al., 2006; Zieger, 2015). Additionally, the landraces used in our experiment were differently sensitive to drought, assessed by the soil water potential at which the plants started reducing their transpiration in a preceding greenhouse experiment (Koehler et al., 2022). We hypothesized that the contrasting drought response might come with differences in the microbial interactions of these varieties.

In summary, our experiment was targeted at understanding how rhizosphere microbiome selection compares for two different landraces of maize by resolving a distance gradient along the radial root axis, including a distal and proximal rhizosphere fraction. To understand the influence of the environment compared to plant-mediated effects, we analyzed this for two field locations differing in edaphic and climatic conditions. The knowledge gained contributes to a better understanding of the most fundamental drivers modulating the rhizosphere and root microbiome and provides baseline information on the microbiome of European maize landraces.

## 2 Materials and methods

### 2.1 Experimental setup

The experimental design of our field experiment was as follows:


sequencingrun×fieldlocation×block(fieldlocation)×

c⁢o⁢m⁢p⁢a⁢r⁢t⁢m⁢e⁢n⁢t×s⁢a⁢m⁢p⁢l⁢i⁢n⁢g⁢t⁢i⁢m⁢e×t⁢r⁢e⁢a⁢t⁢m⁢e⁢n⁢t×v⁢a⁢r⁢i⁢e⁢t⁢y

Table 1 and Supplementary Figure 1 contain additional information on these factors and a summary of the experimental design. Samples originated from a field experiment conducted in 2021, where maize (*Zea mays* L.) was grown at two field locations (factor “field location”) in southern Germany: in Bayreuth and near Ruhstorf an der Rott (hereafter referred to as Ruhstorf) (Supplementary Figure 1B). Locations mainly differed in climatic conditions and the texture and type of the topsoil, sandy loam/ Stagnosol in Bayreuth, and silt loam/ Luvisol in Ruhstorf (Supplementary Figure 2 and Supplementary Table 1). Cumulative precipitation over the growth phase was average compared to historical precipitation in Bayreuth and above average in Ruhstorf (Supplementary Figure 3A). At both field locations, precipitation was below average during the germination and early vegetative growth phase and exceptionally high in Ruhstorf in the weeks before the second sampling phase (see below).

**TABLE 1:** Summary of the factors included in the experimental design.

Factor	Nested in factor	Fixed/random	n levels	Levels	n samples per level
Sequencing run		Random	2	run1, run2	84
Field location		Fixed	2	Bayreuth (bt), Ruhstorf (ru)	84
Block	Field location	Random	2 × 3	bt1, bt2, bt3, ru1, ru2, ru3	28
Compartment		Fixed	4	bulk soil (BS), stripped rhizosphere (RS), washed rhizosphere (RW), root (RT)	BS: 48, RS: 24, RW: 48, RT: 48
Sampling time		Fixed	2	flowering, grain-filling	flowering: 72, grain filling: 96
Treatment		Fixed	2	control, sheltered	84
Variety		Fixed	2	Braunes Schindelmeiser (SC), Gelber Badischer Landmais (GB)	84

We investigated two maize landraces (factor “variety”) originating from Germany (Mayer et al., 2017): Gelber Badischer Landmais (“GB”, obtained from Erfurter Samen und Pflanzenzucht GmbH, Erfurt, Germany) and Braunes Schindelmeiser (“SC”, obtained from the Institute for Crop Science and Plant Breeding of the Bavarian State Research Center for Agriculture (LfL), Freising, Germany). Gelber Badischer Landmais is one of the European landraces used to generate inbred lines that served as parental lines in the development of European hybrid varieties (Messmer et al., 1992). Braunes Schindelmeiser was among the highest-yielding and most popular varieties in eastern Germany in the 1940s and played an important role in the development of inbred and hybrid varieties in the GDR and Soviet Union (Zieger, 2015).

At both field locations, we implemented control (receiving full natural precipitation) and water-reduced treatments (factor “treatment”). For the water-reduced treatment, precipitation was reduced by transparent rain-out shelters, which partially (60%) covered the plot underneath (Supplementary Figures 4A, B). For each precipitation treatment and variety, we repeatedly recorded the soil water potential over the growing season for at least two plots at a depth of 30 cm at the plot center using TEROS21 sensors (METER Environment, Munich, Germany). The measurements indicated drier soil conditions at both field locations and a less pronounced change in response to rain events for the sheltered group (Supplementary Figure 3B), showing the effectiveness of the shelters.

A complete randomized block design with three blocks per field location (factor “block(field location)”) was used (Table 1 and Supplementary Figure 1B). Each block contained one plot (size: 3 by 4 m) per combination of the factors “variety” and “treatment”. Thus, our experiment included three replications per combination of the factors “field location”, “treatment”, “variety”, “sampling time”, and “compartment” which were distributed over three blocks within each field location.

Plants were sown on 21/05/2021 in Bayreuth and on 31/05/2021 in Ruhstorf. Each plot had four rows of plants with 70 cm distance between rows (Supplementary Figure 1C). The planting density was 9–10 plants m^–2^, resulting in a distance of approximately 14 cm between plants within rows. The Supplementary material contains further information on fertilization and herbicide treatments.

We measured mean weight diameter, carbon and nitrogen concentrations, and the mass of rhizosphere soil. Significant differences between field locations and precipitation treatments were discernible (see Supplementary material, Supplementary Figures 5A, 6, and Supplementary Table 2). Further, biomass of shoot, root, and ears, as well as shoot height, total root length, and root diameter, were recorded and differed significantly between field locations but not between varieties (see Supplementary material, Supplementary Figures 5B, 7, and Supplementary Table 3).

### 2.2 Sampling

For each plot, we sampled and pooled material from three randomly selected plants that grew near the plot center (Supplementary Figure 1C). The sampling procedure was based on a protocol by McPherson et al. (2018). Soil blocks with the plant in their center (width = 20 × 20 cm, depth = 30 cm) were excavated. We covered a distance gradient along the radial root axis by sampling four compartments (factor “compartment”): bulk soil (“BS”, here the soil not adherent to roots), a distal rhizosphere soil fraction (“rhizosphere soil stripped”, “RS”), a proximal rhizosphere soil fraction (“rhizosphere soil washed”, “RW”), and the root (“RT”), comprising rhizoplane and root endosphere. BS was removed gently from the roots by manual shaking after loosening it with a spatula. To obtain RS, RW, and RT, we sampled three root pieces per plant (Supplementary Figures 1C, 4C, D). We sampled mature roots, supposing that microbiome composition has reached a relatively stable state here compared to younger root zones characterized by ongoing assembly processes (Rüger et al., 2021). The sampled roots were selected by the following criteria: (1) Roots should be lateral roots emerging from crown roots, (2) originate from 10 to 20 cm below the junction of root and shoot, (3) have the same size class (assessed visually) and (4) have the same maturity level (assessed by the presence of secondary order lateral roots). The samples from all three plants were pooled. After shaking the roots, RS was gained by manually removing the soil that remained attached. Roots with adhering rhizosphere soil were stored for obtaining RW and RT later. All samples were frozen on the day of sampling and stored at **−**80°C until DNA extraction. RW was obtained by washing with saline (0.3 wt.% NaCl in H_2_O) right before DNA extraction. RT was assessed by extracting DNA from the washed roots. BS, RW, and RT were sampled at two times (factor “sampling time”). We expected the microbiome to be comparatively mature and the effects of the precipitation treatment and variety on microbiome assembly to be best detectable at later developmental stages (Walters et al., 2018; Navarro-Noya et al., 2022). Thus, we took samples at the plant developmental stages of flowering (BBCH 63 to 67, approx. 60 days after sowing) and grain filling (median BBCH around 77, approx. 90 days after sowing). RS was sampled only during the grain-filling stage.

### 2.3 16S rRNA amplicon sequencing

Samples were disrupted by bead beating with a TissueLyser II (QIAGEN, Hilden, Germany). DNA was extracted using phenol/chloroform extraction (see Supplementary material). The V4 region of the 16S rRNA region was amplified in triplicate reactions (Supplementary Figure 1D) of 20 μl using the primers 515f (5′-GTGYCAGCMGCCGCGGTAA-3′) (Caporaso et al., 2011) and 806RB (5′-GACTACNVGGGTWTCTAAT-3′) (Apprill et al., 2015) with NEBNext High-Fidelity 2X PCR Master Mix (M0541, New England Biolabs, Ipswich, MA, USA) and with addition of 4 ng bovine serum albumin (Roche Diagnostics, Mannheim, Germany). PCR was performed on a C1000 Touch Thermal Cycler (BioRad, Hercules, CA, USA) with the following conditions: initial denaturation at 98°C for 2 min, 25 repetitions of 10 s at 98°C, 30 s at 55°C, 30 s at 72°C and final extension for 2 min at 72°C. PCR triplicates were pooled thereafter (Supplementary Figure 1D), and samples were randomly assigned to two separate sequencing runs of 84 samples each (factor “sequencing run”). Library preparation and sequencing were done at the Genomics and Bioinformatics KeyLab of the University of Bayreuth, Germany. Amplicons were purified using the NucleoMag 96 PCR purification kit (Machery-Nagel, Düren, Germany) prior to the indexing PCR. Indexing and library preparation were done with the Nextera XT V2 Kit (Illumina, San Diego, CA, USA). Sequencing was performed on the iSeq-100 platform (Illumina) in single-end mode, using 293 cycles. From the extraction on, all steps were also done for a negative control (no sample was added in the extraction step) and a mock community of eleven known bacterial taxa.

### 2.4 Read processing

The quality of raw reads was analyzed using fastqc version 0.11.9 (Andrews, 2010) and MulitQC version 1.12 (Ewels et al., 2016). Forward primer sequences were removed with Cutadapt version 3.7 (Martin, 2011) with default settings, discarding reads without a match. Thereby, primer trimming also acted as an initial quality filtering step. We tested different bioinformatic pipelines for generating a feature matrix, including DADA2 version 1.24.0 (Callahan et al., 2016), USEARCH version 11.0.667 (Edgar, 2010) with UNOISE (Edgar, 2016) and with 97% operational taxonomic unit (OTU) clustering. The two sequencing runs slightly differed in their error profiles, and USEARCH, with 97% OTU clustering, performed best at addressing these batch effects. Therefore, subsequent steps were done with USEARCH commands if not stated differently. Following the recommendations from the USEARCH documentation,^1^ reads from both sequencing runs were processed together. Reads were truncated to 200 nucleotides by removing nucleotides from the 3’-end after assessment of the read quality from quality per read-length diagrams and the output summary of fastq_eestats2. Quality filtering of truncated reads was done with fastq_filter (fastq_maxee = 1.0), followed by dereplication with fastx_uniques. OTU clustering was performed with cluster_otus (minsize = 2). Taxonomy assignment was done with DADA2 using the commands assignTaxonomy and addSpecies with trainsets built on version 138.1 of the SILVA rRNA reference database (McLaren and Callahan, 2021). The USEARCH command otutab was used to generate a sample by OTU matrix. Further data processing was done with phyloseq version 1.40.0 (McMurdie and Holmes, 2013) in R version 4.2.3. OTUs not assigned to prokaryotic taxa (mainly plant chloroplast and mitochondria) were removed and read counts from negative controls were subtracted from read counts in samples.

### 2.5 Data analysis

Statistical analysis was performed with R and PRIMER version 7.0.23 (PRIMER-e, Auckland, New Zealand). To test for differences in rhizosphere soil and plant variables between groups defined by the factors included in the experiment, we calculated Euclidean distances on z-transformed values and used permutational multivariate analysis of variance (PERMANOVA) with sums of squares type III and permutation of residuals under a reduced model in PRIMER (Anderson, 2001). Microbial relative abundance data were rarefied to the number of observations in the sample with the least (6750) observations before analysis of α- and β-diversity. To reduce the weight of highly abundant OTUs, square root and Wisconsin double transformations were applied to the relative abundances before calculating the Bray-Curtis-similarities between samples. Non-metric multi-dimensional scaling (nMDS) and calculation of α-diversity metrics were performed using phyloseq. Kruskal-Wallis tests, followed by Dunn’s post-hoc tests, were used to test for differences in α-diversity metrics between groups. We used PERMANOVA with sums of squares type I and permutation of residuals under a reduced model to test for differences in community structure between experimental groups. Estimates of components of variation in the PERMANOVA output provide an unbiased value for comparing the relative importance of model terms in explaining the total variation (Underwood and Petraitis, 1993). We report the square root of the estimate ($\sqrt{var.}$) as this value has the same unit as the original resemblance measure (Euclidean distance for environmental variables and Bray-Curtis-similarity for community data) (Anderson, 2017). Since the factors “sequencing run” and “block(field location)” were partially confounded, making interaction terms including “block(field location)” inestimable, and since the effect of “sequencing run” was not significant (PERMANOVA, pseudo-F = 107.51, *P* = 0.0879, $\sqrt{var.}$ = 10.15), this factor was omitted in downstream analysis and the initial PERMANOVA test was repeated without it. Using a simplified design including only significant factors (*P* < 0.05), pairwise comparisons between levels of factors were performed using PERMANOVA. For the comparison among levels of the factor “field location”, a low number of possible unique permutations reduced the power of the test. Therefore, here, P-values were additionally computed using Monte-Carlo tests (Anderson and Robinson, 2003). Differences in dispersion, representing differences in multivariate variability, which can be interpreted as a measure of β-diversity (Anderson et al., 2006), were analyzed with PERMDISP on distances to centroids of groups in PRIMER. To identify ubiquitous taxa and potential general patterns in their change of relative abundance, we analyzed core microbiomes. For this, we used the most conservative definition of a core microbiome (Neu et al., 2021): An OTU had to appear in all samples of a group (100% occupancy) to be considered a member of the core microbiome. We used non-rarefied data for this analysis as recommended by (Neu et al., 2021). For differential abundance analysis, taxa from the full dataset were agglomerated at the lowest taxonomic level with bootstrap support > 50% as determined by DADA2s assignTaxonomy command using the phyloseq function tax_glom. We identified differentially abundant taxa within field locations between compartments using the function ancombc2 from the R package ANCOMBC (version 2.4.0) (Lin and Peddada, 2020). ANCOMBC is tailored to the characteristics of community data (Lin and Peddada, 2020) and is considered conservative, meaning that it likely has a low false positive rate (Nearing et al., 2022). Since PERMANOVA showed that blocks explained a significant proportion of the variance in the data, “block” was included as a random effect in the linear mixed models used by the ancombc2 function. Figures were generated with ggplot2 version 3.4.1 (Wickham, 2016) in R.

## 3 Results

### 3.1 Effect of field location, compartment, sampling time, treatment, and variety on the prokaryotic community

**FIGURE 1:**
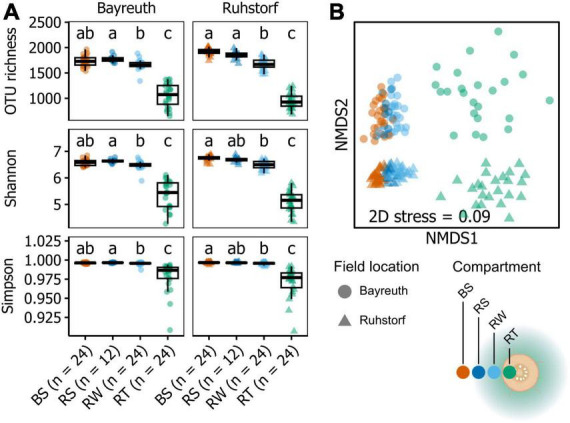
**(A)** Alpha diversity indices shown by field location and compartment (bulk soil: BS, stripped rhizosphere: RS, washed rhizosphere: RW, root: RT). Significant differences (*P* < 0.05), as determined by Dunn’s tests, are indicated by the compact letter display. Groups not sharing a letter are significantly different from one another. **(B)** nMDS ordination based on Bray-Curtis-similarities between samples calculated on relative abundances of OTUs after rarefaction, square root transformation, and Wisconsin double transformation. Each point represents a sample, and the distance between points reflects the dissimilarity of their communities.

**FIGURE 2:**
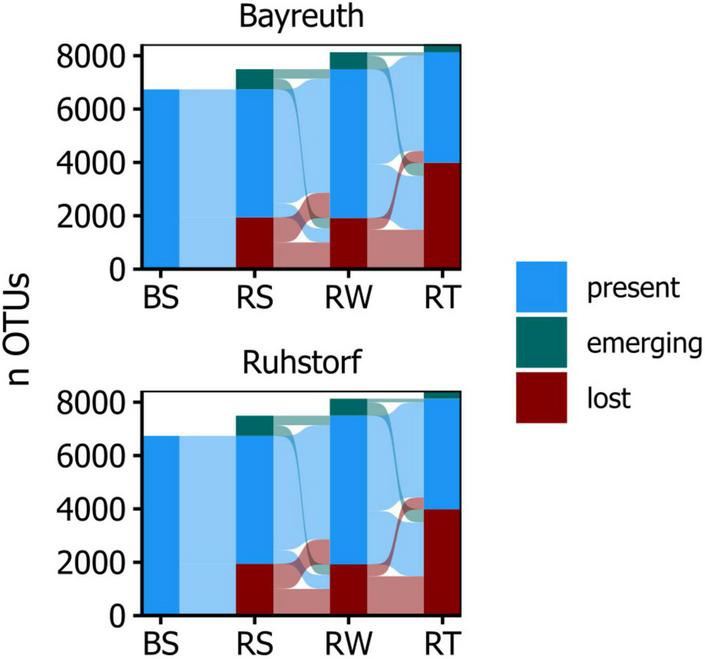
Alluvial diagram showing the number of OTUs per compartment that are passed on, lost, or emerging, comparing adjacent compartments along the radial root axis.

Community composition in 168 samples was assessed by metabarcoding to investigate how the prokaryotic community changed with proximity to the root, how this differed between field locations, and how the factors sampling time, variety, and treatment (water availability) influenced communities. Amplicon sequencing yielded 11286 OTUs in total after all filtering steps. Sample coverage ranged from 0.92 to 0.99, with a tendency toward higher coverage from soil to root samples at the field location near Ruhstorf (Supplementary Figure 8). The α-diversity within compartments was similar at both field locations (Figure 1A). With increasing proximity to the root, the number of taxa in prokaryotic communities (richness) decreased significantly (Supplementary Table 4). At the same time, communities became increasingly dominated by fewer taxa (expressed through Shannon and Simpson indices). Variability of α-diversity measures also increased toward the root. These patterns were consistent for both field locations. Viewed from the bulk soil toward the root, the OTUs in a compartment predominantly represented subsets from the community of the preceding compartment (Figure 2). Only a few OTUs emerged per compartment that were not observed in the preceding one.

Community composition corresponded significantly to field location (PERMANOVA, pseudo-F = 9.1768, *P* = 0.0043, $\sqrt{var.}$ = 22.07) (Figure 1B and Supplementary Table 5) and compartment (PERMANOVA, pseudo-F = 10.922, *P* = 0.0001, $\sqrt{var.}$ = 21.96), representing the distance gradient toward the root. The relative importance of the factors field location and compartment was similar. We further found block effects (PERMANOVA, pseudo-F = 2.9289, *P* = 0.0005, $\sqrt{var.}$ = 10.39), which mainly affected the field location near Bayreuth (Supplementary Figure 9). Significant interaction between the factors field location and compartment (PERMANOVA, pseudo-F = 2.5939, *P* = 0.0014, $\sqrt{var.}$ = 12.37) indicated differences in the change of community composition among compartments between field locations. All other factors (sequencing run, sampling time, treatment, and variety) had no significant effects on overall community composition. For further analysis, we thus focused only on the significant factors compartment and field location. Using pairwise comparisons, we could show that communities significantly differed between field locations in all compartments (Figure 1B, PERMANOVA results in Supplementary Table 6). Comparing compartments within each field location, we showed that at both field locations, all compartments were significantly different from one another except for the BS and RS compartments at the field location near Bayreuth (Figure 1B, PERMANOVA results in Supplementary Table 7). Further, dispersion differed between field locations and compartments (PERMDISP, pseudo-F = 50.982, *P* = 0.0001, Figure 1B and Supplementary Table 8). Dispersion between compartments within field locations varied for comparisons between the BS, RS, and RW compartments with the RT compartment (Supplementary Table 8). Considering the results from PERMANOVA and PERMDISP together with the positioning of the samples in the NMDS ordination (Figure 1B), we can infer that the significant differences between field locations and compartments were both due to directional shifts in community composition and significantly increased β-diversity in the RT compartment.

### 3.2 Abundance of core microbiome members

**FIGURE 3:**
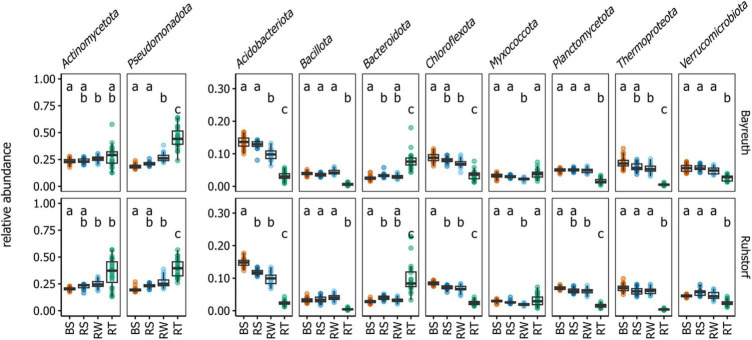
Relative abundances of the overall most abundant phyla by field location and compartment. Significant differences (*P* < 0.05), as determined by Dunn’s tests, are indicated by the compact letter display. Groups not sharing a letter are significantly different from one another.

**FIGURE 4:**
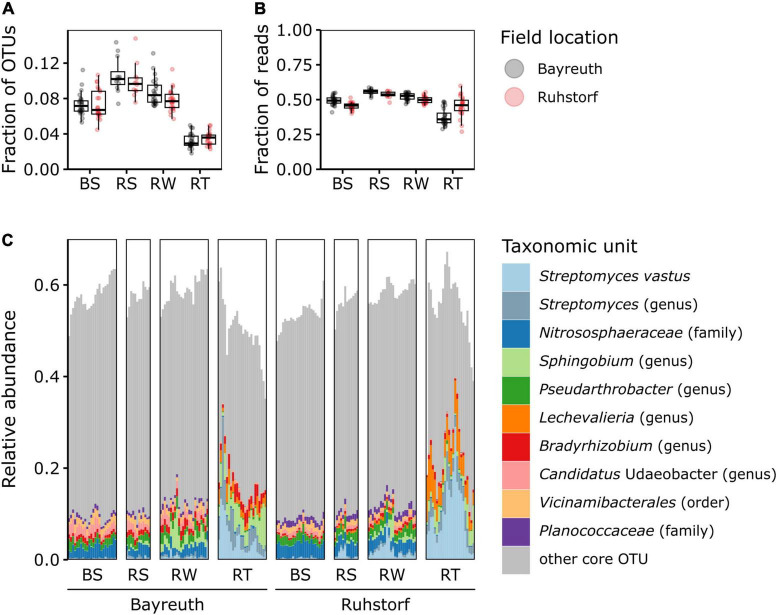
OTUs of the core microbiome (OTUs shared by all samples of a compartment across both field locations). **(A)** Proportion of core OTUs out of all OTUs shown by field location and compartment. Note that the range of theoretically possible values of the ordinate is 0 to 1, but only a section is shown to visualize differences between groups better. **(B)** Proportion of reads belonging to core OTUs out of all reads shown by field location and compartment. **(C)** Relative abundances of core OTUs by field location and compartment. The ten most abundant taxonomic groups are colored according to the lowest taxonomic level with bootstrap support (minBoot = 50) from DADA2s assignTaxonomy command. Note that the range of theoretically possible values of the ordinate is 0 to 1, but only a section is shown to visualize differences between groups better.

To identify potential general patterns in relative abundance shifts between the investigated compartments, we first compared microbiome patterns at the phylum level. Abundant taxa mainly belonged to *Pseudomonadota*, *Actinomycetota*, and *Acidobacteriota* (Figure 3 and Supplementary Figure 10). Differences between compartments were clearly apparent. We further focused on OTUs constituting the core microbiome. The OTUs present in all samples of a compartment across both field locations only comprised a small fraction of all OTUs (between 1.3 and 11.0%, mean = 5.2%, Figure 4A) but accounted for a large fraction (between 24.8 and 55.8%, mean = 43.6%, Figure 4B) of all reads. The three most abundant taxa over all compartments belonged to *Streptomyces* spp., the *Nitrososphaeraceae*, and *Sphingobium* sp. (Figure 4C). Noticeable was an increase in the abundance of *Streptomyces* spp., *Sphingobium* sp., and *Lechevalieria* sp. with increasing proximity to the root. Almost all other core OTUs with high relative abundance in the BS, RS, and RW compartments had a lower relative abundance within the RT compartment. The relative abundance of *Nitrososphaeraceae*, which dominated the community in BS samples and had a high relative abundance in RS and RW, decreased markedly toward the root. Comparing core microbiome members for the individual field locations, precipitation treatments, and varieties (Supplementary Figure 11) showed that about two-thirds of the core taxa were shared among sites, and one-third was specific to either field location. No apparent differences in the number and identity of the core taxa were detectable for comparisons between the control and sheltered treatments and varieties across and within field locations. Also, for the core microbiome, strong effects of the compartment type on the number of core taxa and their shift in relative abundance were apparent. These occurred consistently at both field locations.

### 3.3 Differential abundance along the radial root axis

**FIGURE 5:**
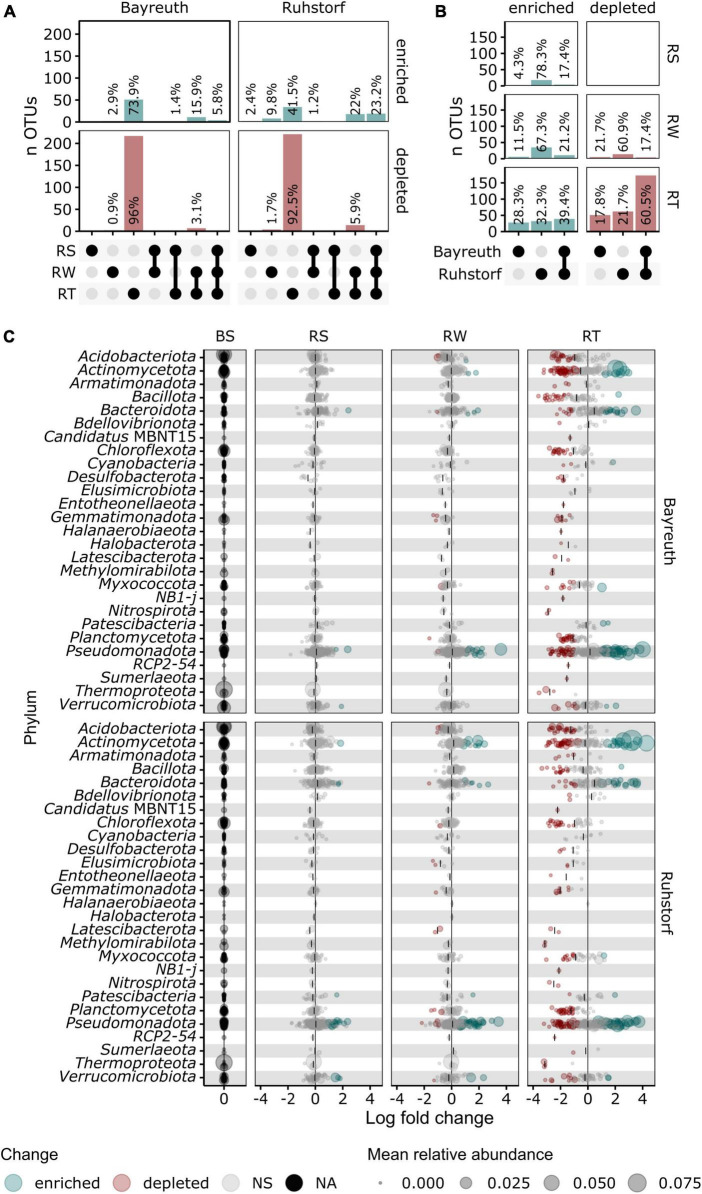
**(A,B)** Upset plots showing proportions of significantly enriched or depleted taxa determined by differential abundance testing with ANCOMBC. **(A)** Proportions by compartment shown for both field locations. **(B)** Proportions by field location shown for all compartments. **(C)** Change in relative abundance compared to bulk soil for taxa grouped by phylum shown for both field locations and each compartment as determined by ANCOMBC. Significantly enriched taxa (alpha < 0.05, log fold change > 0) are shown in green and significantly depleted (alpha < 0.05, log fold change < 0) taxa are shown in red. The median log fold change is indicated by vertical lines.

**FIGURE 6:**
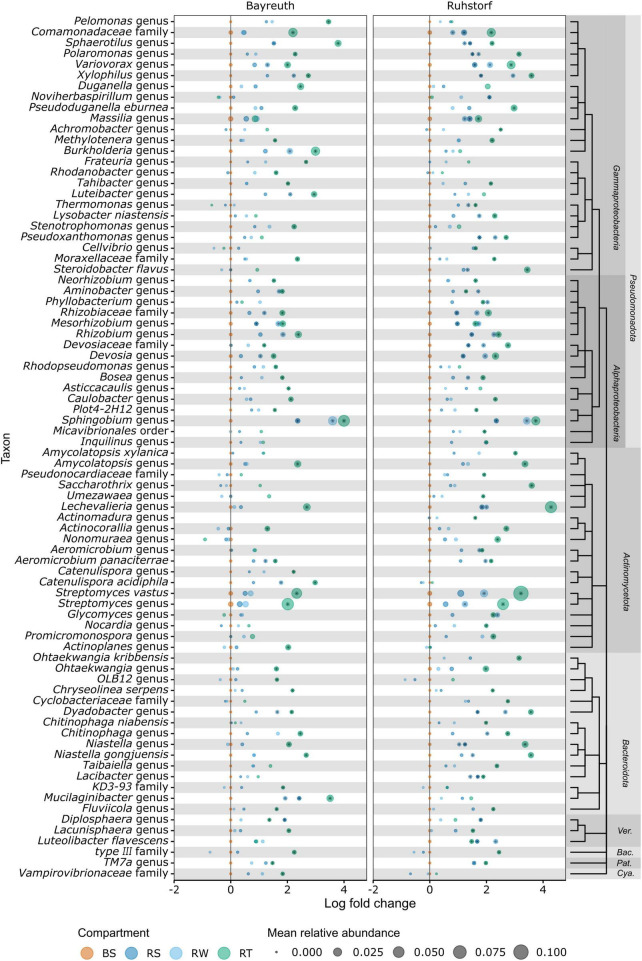
Information on the 80 OTUs showing the strongest enrichment compared to bulk soil (highest log fold change) as determined by ANCOMBC. Taxonomic information is given at the lowest taxonomic level with bootstrap support (minBoot = 50) from DADA2s assignTaxonomy command. Taxonomic relationships are shown by a cladogram. Asterisks indicate significant (alpha < 0.05) enrichment. Ver., *Verrucomicrobiota*; Bac., *Bacillota*; Pat., *Patescibacteria*; Cya., *Cyanobacteria*.

**FIGURE 7:**
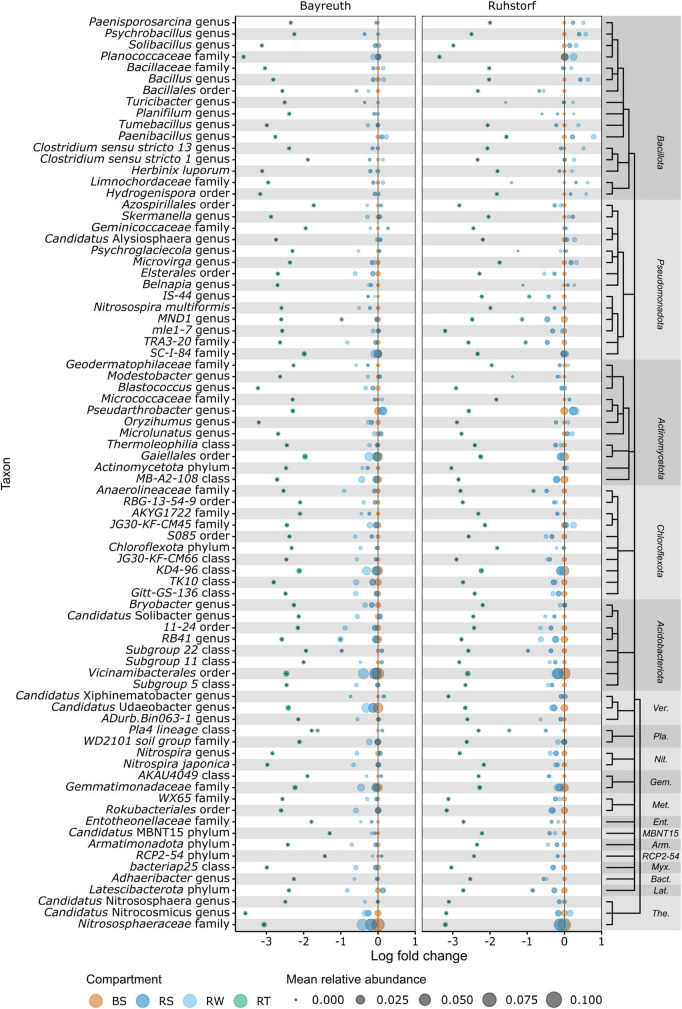
Information on the 80 OTUs showing the strongest depletion compared to bulk soil (highest negative log fold change) as determined by ANCOMBC. Taxonomic information is given at the lowest taxonomic rank with bootstrap support (minBoot = 50) from DADA2s assignTaxonomy command. Taxonomic relationships are shown by a cladogram. Asterisks indicate significant (alpha < 0.05) enrichment. Ver., *Verrucomicrobiota*; Pla., *Planctomycetota*; Nit., *Nitrospirota*; Gem., *Gemmatimonadota*; Met., *Methylomirabilota*; Ent., *Entotheonellaeota*; Arm., *Armatimonadota*; Myx., *Myxococcota*; Bact., *Bacteroidota*; Lat., *Latescibacterota*; The., *Thermoproteota*.

To identify the taxa accounting for the observed patterns in community composition between compartments, we compared relative abundances at the lowest taxonomic level with reliable taxonomic information by differential abundance testing. Of 1331 taxa, 401 (30.1%) were identified as differentially abundant between compartments. Their number strongly increased with proximity to the root (Figure 5A). In the RS and RW compartments, most differentially abundant taxa were enriched. In contrast, most differentially abundant taxa were depleted in the RT compartment. Most taxa enriched in RS and RW were also enriched in the RT compartment, whereas few were enriched specifically in RS and RW. Patterns of enrichment were consistent across both field locations (Figures 5B, C). The total number of differentially abundant taxa was larger in Ruhstorf (Figure 5B). In the RT compartment, 39.4% of all significantly enriched and 60.5% of all significantly depleted taxa were enriched or depleted at both field locations. Depleted taxa belonged to almost all phyla, while enriched taxa mainly belonged to the *Actinomycetota*, *Bacteroidota*, *Pseudomonadota*, and *Verrucomicrobiota* (Figure 5C). With few exceptions, taxa significantly enriched in the RT compartment also showed a trend toward higher relative abundance in RS and RW (Figure 6). Particularly strong enrichment was observed for *Sphingobium* sp., which was highly enriched across all compartments and at both field locations. Also, specific taxa belonging to the *Actinomycetota*, especially *Streptomyces* spp. and *Lechevalieria* sp., were highly enriched compared to BS, with *Lechevalieria* sp. showing the strongest enrichment of all taxa in Ruhstorf. Most significantly depleted taxa in the root also showed a trend toward depletion in the rhizosphere compartments (Figure 7). An exception were members of the *Bacillota* and *Pseudomonadota*, many of which showed a trend toward enrichment in RS and RW, at least at the field location near Ruhstorf, but were significantly depleted in the RT compartment. Taxa with high relative abundance in BS that were significantly depleted included the order *Vicinamibacterales*, *Ca.* Udaeobacter, and archaea from the family *Nitrosospheraceae*, which are known as ammonia-oxidizers. Also, bacteria involved in nitrification, such as *Nitrosospira multiformis* and members of the *Nitrospira* genus, were significantly depleted toward the root. Usually, stronger enrichment or depletion was observed in the proximal rhizosphere compartment gained by washing (RW) compared to the distal rhizosphere compartment gained by manual stripping (RS). For most enriched or depleted taxa, the magnitude and mode of the change were consistent between field locations (Figures 6, 7). Further, the majority of the enriched and depleted taxa were members of a core microbiome (Supplementary Figure 11).

## 4 Discussion

### 4.1 Field location and proximity to the root shaped the prokaryotic community

The analysis of samples representing distance gradients along the radial root axis of *Z. mays* from two field locations, differing in multiple environmental factors, allowed us to compare the relative importance of the field location to the plant-mediated effect on prokaryotic community composition. Both field location and proximity to the root had a similarly strong effect (Figure 1B and Supplementary Table 5). Diverging estimates on the influence of environmental factors compared to plant-mediated effects have been previously reported. Xiong et al. (2021) found the impact of compartment niche to be much stronger than the effect of environmental factors, including soil type. In contrast, Peiffer et al. (2013) described a stronger association with field location for rhizosphere soil. Together with our results, this shows a high context dependency of the relative importance of field location-associated environmental factors compared to plant-mediated factors in their influence on microbiome composition. Especially pH (Lauber et al., 2009), nitrogen, and organic carbon content (Cederlund et al., 2014), soil moisture (Serna-Chavez et al., 2013; Naylor et al., 2023), and redox status (DeAngelis et al., 2010) are key factors explaining variation in biogeographic patterns of soil microbiomes (Fierer, 2017). We thus assume that the differing edaphic and climatic conditions (Supplementary Figures 2, 3, 5A, 6 and Supplementary Table 2) probably contributed strongest to the patterns in prokaryotic community composition observed between field locations in our experiment.

While the patterns in community composition corresponding to proximity to the root and the field location were marked (Figure 1B and Supplementary Table 5), we could not detect significant differences associated with the factors sampling time, treatment (water availability), and maize variety (Supplementary Table 5). Samples were taken during the flowering and grain-filling developmental stages, representing a time difference of about 30 days. It has been shown that microbiome composition changes dynamically throughout the life cycle of plants (Edwards et al., 2018; Walters et al., 2018). However, this change is strongest during the vegetative phase, and the formation of a mature, less dynamic microbiome has been described for the reproductive stage of maize (Bai et al., 2022). Contrastingly, also observations of increasing bacterial diversity in later plant developmental stages exist, suggesting reduced plant control on microbiome composition (Bourceret et al., 2022; Navarro-Noya et al., 2022). Accordingly, it has been suggested that plants reduce their investment into root exudation and interaction with the microbiome with the onset of the reproductive phase (Bonkowski et al., 2021). We posit that potential shifts in community composition during the reproductive phase were too small to be detected in our setting within the relatively short period covered between sampling events. Resolving the potential influence of water availability and maize variety on the microbiome for the vegetative growth phase could be an objective of future work.

We observed minor differences in rhizosphere soil properties between water availability treatments, mainly attributable to varying amounts of obtainable rhizosphere soil (Supplementary Figures 5A, 6 and Supplementary Table 2). However, a response of the microbial community to water availability was not detectable for any of the analyzed compartments. The year of our experiment was characterized by a high amount of precipitation, especially during the reproductive growth phase in August (Supplementary Figure 3A). Therefore, although the rain-out shelters effectively reduced precipitation compared to the control group (Supplementary Figure 3B), the conditions for the sheltered group were probably still moist in contrast to other studies analyzing drought effects on soil microbiomes. A shift in microbiome composition corresponding to water availability as described by others (e.g., Wang et al., 2020; Naylor et al., 2023) may simply not have occurred under the comparatively moist conditions in our experiment.

The varieties analyzed here had shown contrasting responses to a drought treatment in a preceding greenhouse experiment (Koehler et al., 2022). In our field experiment, we could not detect differences in their microbiome (Supplementary Table 5) and plant variables (Supplementary Table 3). Koehler et al. (2022) compared the response of the varieties to soil drying and a differing response does not necessarily imply differences in basic parameters such as plant biomass. Association of microbiome composition and plant varieties or genotypes has been previously reported (Bouffaud et al., 2012; Peiffer et al., 2013; Xiong et al., 2021), however, the effects were usually weak. Especially for intraspecific comparisons, their detection in a field setting might require a much larger number of replications than was implementable in our experiment (Walters et al., 2018). Our observation of increasing variation in microbiome composition with proximity to the root indicates a comparatively high intra-variety variation in the investigated landraces, which would hamper the detection of differences between varieties. In addition, the overall water availability in our field experiment was higher than in the preceding greenhouse experiment, so that potential variety effects occurring under very dry conditions would not have been detected. We conclude that the contrasting drought response observed for the varieties in the greenhouse did not come with an effect on the microbiome detectable in our field setting. However, testing this under more severe drought and with more replicates would be relevant.

From our experiment, we infer that potential differences induced by sampling time, water availability, and maize variety were much weaker than those corresponding to compartment and field location. Since the differences we detected between compartments stand out clearly and because many patterns were observable consistently across both field locations, we suppose that our observations on the shift in microbiome composition along the radial root axis are of broad applicability. We will thus focus on this in the further discussion.

### 4.2 Specialization of the community toward the root

Also, for European maize landraces, we revealed the previously described (Peiffer et al., 2013; Edwards et al., 2015; Walters et al., 2018; Xiong et al., 2021; Bai et al., 2023) pattern that microbial communities between bulk soil, rhizosphere, and roots are highly distinct and decrease in taxonomic richness toward the root (Figure 1 and Supplementary Table 5). Further, we resolved differences between the proximal and distal rhizosphere. Differences in microbiome composition between rhizosphere compartments have been reported for maize (Brisson et al., 2019), but the definition of the proximal rhizosphere used in that study encompasses a much larger soil volume than in our work. Although the general patterns we observed for both rhizosphere compartments were similar, some patterns could only be ascertained from the proximal rhizosphere (Figure 5). Thus, our results demonstrate that considering the extension of the rhizosphere is of great importance in microbiome studies. Already minor methodological differences in the sampling procedure used to obtain rhizosphere soil can significantly influence findings.

The identity of the taxonomic groups most affected by the reduction in diversity and, especially, of those taxa gaining relative abundance toward the root was largely consistent across replicates (Figures 3–5). Further, these patterns were consistent across a range of experimental conditions, including different field locations, sampling times, watering treatments, and landrace varieties. Also, focusing on the core microbiome, which comprised a large number of taxa with a comparatively high relative abundance, clear shifts in microbiome composition with proximity to the root were discernible (Figure 4 and Supplementary Figure 11). These observations are in line with the assumption that the community in the rhizosphere and root constitutes specialized subsets of the taxa present in the surrounding bulk soil, well adapted to the conditions in the microenvironments within these compartments (Reinhold-Hurek et al., 2015). Additionally, the influence of environmental factors has been described to decrease with increasing proximity to the root, meaning that root and rhizosphere communities from distinct geographical locations are more similar to each other than bulk soil communities from the same locations (Thiergart et al., 2019). However, also a stronger effect of soil texture on the prokaryotic and protistan communities in the rhizosphere of maize compared to bulk soil has been shown (Rüger et al., 2023). We did not find an increased similarity in overall community composition with increasing proximity toward the root when comparing compartments between field locations. Instead, we observed higher variation in α-diversity and higher β-diversity in the root compartment compared to samples of the soil compartments (Figure 1). This has been shown for the comparison between bulk and rhizosphere soil, rhizoplane, and endosphere of multiple plant species (Peiffer et al., 2013; Thiergart et al., 2019). However, since the root compartment should be characterized by comparably stable conditions induced by the impact of the plant immune system (Bulgarelli et al., 2013), the observation seems unexpected. A potentially larger role of random and priority effects in the root compartment could explain this pattern (Fitzpatrick et al., 2020). Additionally, landraces are characterized by higher genetic and phenotypic intra-variety variation compared to the genetically uniform inbred and hybrid lines used in most studies (Pérez-Jaramillo et al., 2016; Schmidt et al., 2016). Since the influence of plant effects should be most pronounced for the RT compartment, it seems plausible that the high variation in microbiome composition could be a consequence of variation in plant traits between landrace individuals.

In summary, our findings and those of others (Nuccio et al., 2016; Xiong et al., 2021) show that for certain taxonomic groups, the effect of compartment niche can dominate over the effect of field location-associated environmental factors even when comparing locations that differ in multiple environmental factors, including soil texture and precipitation. We suggest that the consistent changes in relative abundance across field locations observed here at high taxonomic levels (phylum) and for individual genus-level taxa can be seen as a strong indicator for the relevance of the respective taxa from a host perspective.

### 4.3 Identification of microbiome members with potential plant-relevance

We reveal differential abundance patterns for a large number of taxa with high taxonomic resolution (Figures 6, 7). Here, we focus on taxa that dominated communities or showed particularly strong abundance shifts but point out that also rare taxa can have highly relevant functions (Jousset et al., 2017). The taxa discussed here were also members of the core microbiome, occurring in all samples across both field locations (Supplementary Figure 11). Genus-level taxa with remarkable enrichment and dominance in our setting were *Sphingobium* sp. (*Alphaproteobacteria*), *Streptomyces* spp., and *Lechevalieria* sp. (both *Actinomycetota*) (Figure 6). *Sphingobium* sp. was previously found to dominate rhizosphere communities and to show enrichment toward roots of different *Poaceae* [*Ammophila breviligulata* L. (Boss et al., 2022), *Panicum virgatum* L. (Singer et al., 2019) and *Z. mays* (Peiffer et al., 2013; Bai et al., 2023)]. Members of the genus *Sphingobium* have been described in the context of plant growth promotion (Boss et al., 2022). However, inferring functional roles from taxonomic information at the genus or even higher taxonomic levels is challenging since many genera are known to comprise mutualists, commensals, and pathogens. Generally, the *Pseudomonadota* are considered to be fast-growing r-strategists, with their abundance fluctuating with carbon substrate availability (Fierer et al., 2007). This might constitute a competitive advantage within the rhizosphere and roots. *Streptomyces* spp. are well known as common and abundant members of soil microbiomes and for living in symbiosis with many plant species, also as endophytes (Vurukonda et al., 2018; Singer et al., 2019; Bai et al., 2023). Members of the genus have been investigated for their plant-growth-promoting properties and are known to control other microorganisms by producing antibiotics (Vurukonda et al., 2018). For *Lechevalieria* sp., an enrichment by nitrogen-inefficient maize hybrids (Li et al., 2023), under drought (Ma et al., 2022), and under nitrate fertilization (Mang et al., 2023) has been described. Taken together, these findings suggest that both investigated landraces, independent of soil or field location, had a notable capacity to enrich for bacterial taxa which might have functions relevant from a plant perspective.

We further identified several taxa that showed reduced abundance with increasing proximity to the root or were enriched in the rhizosphere but depleted in the root (Figure 7). The latter pattern was particularly pronounced for members of the *Bacillota* in Ruhstorf, for example, *Paenobacillus*, a genus that is well recognized to occur in the rhizosphere and that includes members with several functions that are considered beneficial for plants (Grady et al., 2016). A mechanism causing depletion would be outcompetition by taxa with better adaptation to the conditions in the rhizosphere and root compartments. *Vicinamibacterales* were among the dominant taxa in bulk soil and showed a strongly reduced abundance toward the root. Members of this order have recently been described for their potential role in phosphate solubilization (Wu et al., 2021). *Ca.* Udaeobacter (belonging to the *Verrucomicrobiota*) is recognized as one of the most prevalent bacterial taxa in soil and has been shown to successfully cope with high concentrations of antibiotics (Willms et al., 2020). Still, its potential role in the rhizosphere remains unknown. Most remarkable was an almost total depletion of ammonia-oxidizing archaea (AOA) from the *Nitrosospheraceae* family and of the ammonia-oxidizing bacteria (AOB) *Nitrosospira multiformis* and members of the *Nitrospira* genus in the root compartment observed in our study. This contradicts the observation by Wattenburger et al. (2020), who found an increase in AOA abundance in the rhizosphere of maize. We suppose that the depletion could be explained by indirect competition for ammonium with the plant or by direct active suppression by the plant in competition for ammonium. For many plant species, the production of biological nitrification inhibitors (BNIs), secondary metabolites that inhibit nitrification in the rhizosphere, has been described (Coskun et al., 2017). BNIs might explain the observation that the abundance of AOB in soil increases after ammonium addition but that this increase is less pronounced in the rhizosphere, which has been made for barley (Glaser et al., 2010). Recently, the first BNIs were identified for maize (Otaka et al., 2022). Nitrification inhibition could be a trait lost in modern maize varieties bred under high nutrient supply and for use in high-input agricultural systems. For wheat (*Triticum aestivum*), it has been shown that modern varieties exude insufficient amounts of BNIs compared to wild relatives (Subbarao et al., 2007) and multiple landraces (O’Sullivan et al., 2016), and BNI production has been introduced into modern varieties after identification of the responsible chromosome region (Bozal-Leorri et al., 2022).

## 5 Conclusion

Our observations provide a detailed perspective of prokaryotic communities across rhizosphere and root compartments of two landraces of *Z. mays* grown at two distinct field locations. Although communities were differentiable between field locations, about two-thirds of the taxa occurring in all samples were shared across field locations. We further found clear abundance shifts with proximity to the root occurring consistently across both field locations. Potential effects of water availability, plant variety, and time after planting were not discernible in our data, indicating a comparably low influence. This highlights the need for larger sample numbers to investigate the role of such factors under field conditions. We identified various taxa that were either enriched or depleted toward the root. The consistent observation of these patterns across both field locations suggests that the affected taxa could be relevant for the plant. Future work should further characterize their specific functions and interactions with the plant. A profound understanding of the mechanisms controlling microbiome composition is crucial for integrating microbiome-related traits into breeding and farming practices. Our results contribute to this by providing criteria for the selection of taxonomic groups deserving particular attention.

## Data availability statement

The datasets presented in this study can be found in online repositories. The names of the repository/repositories and accession number(s) can be found below: https://www.ebi.ac.uk/ena, study accession number PRJEB72432 (sample accession numbers ERS18275189 – ERS18275362).

## Author contributions

NT: Writing – original draft, Writing – review and editing, Conceptualization, Investigation, Visualization, Methodology. TK: Writing – original draft, Writing – review and editing, Conceptualization, Investigation. FS: Writing – original draft, Writing – review and editing, Conceptualization, Investigation. S-YT: Writing – original draft, Writing – review and editing, Conceptualization, Investigation. AW: Writing – original draft, Writing – review and editing, Conceptualization, Investigation. AC: Writing – original draft, Writing – review and editing, Conceptualization, Funding acquisition. CM: Writing – original draft, Writing – review and editing, Conceptualization, Funding acquisition. AV: Writing – original draft, Writing – review and editing, Conceptualization, Funding acquisition. SW: Writing – original draft, Writing – review and editing, Conceptualization, Funding acquisition. JP: Writing – original draft, Writing – review and editing, Conceptualization, Funding acquisition, Project administration. TL: Writing – original draft, Writing – review and editing, Conceptualization, Funding acquisition, Supervision.

## Footnotes

1
www.drive5.com/usearch/manual/
