# Supplementary material for: Consistent prokaryotic community patterns along the radial root axis of two Zea mays L. landraces across two distinct field locations
Source: Front Microbiol. 2024 Jul 17;15:1386476. doi: 10.3389/fmicb.2024.1386476 (PMC11292614; doi:10.3389/fmicb.2024.1386476)
Supplement: Supplementary file 1 [file Data_Sheet_1.pdf]

Consistent prokaryotic community patterns along the radial root axis of two *Zea mays* L. landraces across two distinct field locations

Nicolas Tyborski, Tina Koehler, Franziska A. Steiner, Shu-Yin Tung, Andreas J. Wild, Andrea Carminati, Carsten W. Mueller, Alix Vidal, Sebastian Wolfrum, Johanna Pausch, Tillmann Lueders

Frontiers in Microbiology

Supplementary Material

1 Supplementary methods description 1
1.1 Site management 1
1.2 DNA extraction 2
2 Supplementary results 2
2.1 Rhizosphere soil properties 2
2.2 Plant-related variables 2
3 Supplementary Figures and Tables 3
3.1 Supplementary Figures 3
3.2 Supplementary Tables 13
4 Literature cited in the Supplementary Material 17

1 Supplementary methods description

1.1 Site management

Plants were grown using conventional agricultural practices adapted to the local conditions at the field locations. In Bayreuth, the mineral fertilizer ALZON (SKW Stickstoffwerke Piesteritz GmbH, Lutherstadt Wittenberg, Germany) was applied 21 days after sowing (150 kg N ha^{-1}). In Ruhstorf, sludge was plowed into the soil one month before sowing. The mineral fertilizer YaraBela SULFAN (YARA GmbH & Co. KG, Dülmen, Germany) was applied one week after sowing (80 kg N ha^{-1}). In Bayreuth, the herbicides Bromotril 225EC (ADAMA Agricultural Solutions, Ashdod, Israel) and MaisTer power (Bayer CropScience Deutschland GmbH, Monheim, Germany) were applied seven weeks after sowing. In Ruhstorf, Laudis Aspekt Pack (Bayer CropScience Deutschland GmbH, Monheim, Germany) was applied two weeks after sowing.

1.2 DNA extraction

For bead-beating of bulk and rhizosphere soil, samples were placed in 2 ml tubes containing 0.2 ml zirconia beads (diameter = 0.1 mm), 0.2 ml silica beads (diameter = 0.7 mm), and 800 µl lysis buffer (700 µl 112.8 mM Na_2HPO_4 (Carl Roth GmbH + Co. KG, Karlsruhe, Germany), 7.2 mM NaH_2PO_4 (Sigma-Aldrich, Saint Louis, MO, USA), 71.1 mM TRIS hydrochloride (Roth), 54.5 mM Trizma-Base (Sigma-Aldrich), 58.44 mM NaCl (Roth) in H_2O, pH = 8, mixed with 100 µl 20 vol.% sodium dodecyl sulfate solution (Sigma-Aldrich)). Root samples were placed in 2 ml tubes after washing in saline and frozen in liquid nitrogen. Each tube contained 2 stainless steel beads (diameter = 5 mm). Bead-beating settings were 1 min at 30 Hz. For root samples, lysis buffer was added directly after bead-beating, the homogenized tissue was dispersed, and samples were incubated for 5 min. Lysis buffer was discarded after centrifugation, and DNA was extracted subsequently with phenol-chloroform-isoamyl alcohol (PCI, 25:24:1 (vol./vol./vol.), Roth) and chloroform-isoamyl alcohol (CI, 24:1 (vol./vol.), Roth). DNA was precipitated in polyethylene glycol solution (300 g L^{-1} PEG6000 (Roth) and 1.60 M NaCl (Roth) in H_2O) by incubation for 1 h at 4 °C followed by centrifugation at 4 °C for 30 min. We washed the resulting pellet with chilled (–20 °C) 80 vol.% ethanol. The pellet was then dried for 5 min and subsequently solved in 50 µl elution buffer (QIAGEN, Hilden, Germany). All centrifugation steps were performed for 5 min at 16,000 × g at 4 °C. For PCR, extracts were diluted to 10 ng $µl^{-1}$.

2 Supplementary results

2.1 Rhizosphere soil properties

Physical and chemical soil properties in the rhizosphere differed significantly between field locations (PERMANOVA, pseudo-F = 38.658, P(Monte-Carlo) = 0.0001, full results in Supplementary Table 2), with a higher C and N content in microaggregates in Bayreuth and in macroaggregates in Ruhstorf (Supplementary Figures 5A and 6). A weak but significant difference between sheltering treatments (PERMANOVA, pseudo-F = 3.964, P = 0.0293) was apparent.

2.2 Plant-related variables

Plant-related variables differed significantly between field locations (PERMANOVA, pseudo-F = 38.658, P(Monte-Carlo) = 0.0021, full results in Supplementary Table 3). Aboveground biomass was higher, and plants were taller in Ruhstorf, while plants in Bayreuth had a larger total root length and, at the same time, smaller mean root diameters (Supplementary Figures 5B, 7).

3 Supplementary Figures and Tables

3.1 Supplementary Figures

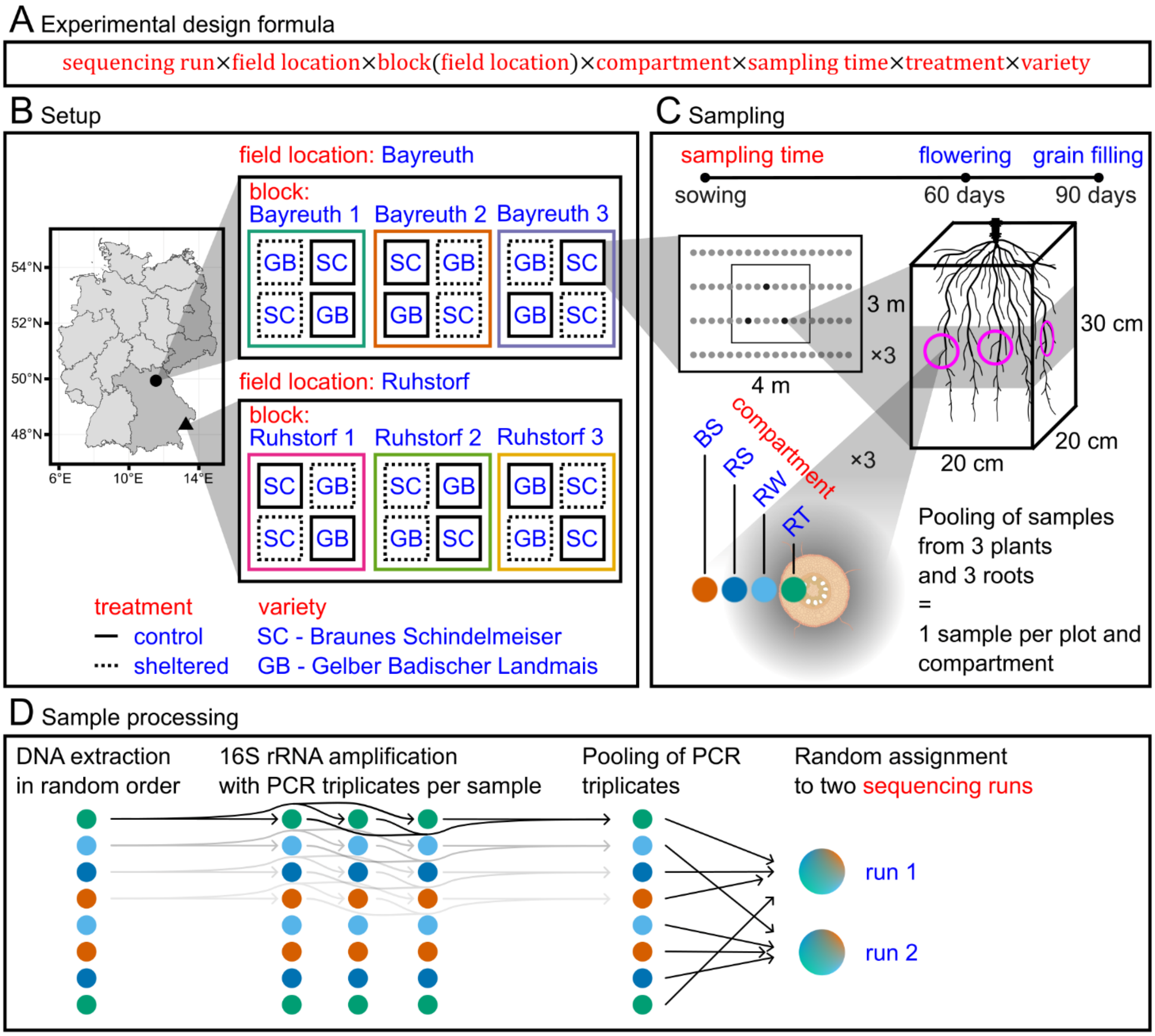

Supplementary Figure 1. Summary of the experimental design. Experimental factors are shown in red letters, and levels of experimental factors are shown in blue letters. **(A)** Experimental design formula. The symbol × indicates that experimental factors were crossed with each other. The brackets indicate that blocks were nested within field locations. **(B)** The experiment was replicated at two sites (Bayreuth and Ruhstorf) in Bavaria, Germany. The map shows the geographic positions of the field locations. At each field location, we set up three replicated blocks. Each block contained one plot per combination of the factors treatment (with levels control and sheltered) and variety (with levels Braunes Schindelmeiser and Gelber Badischer Landmais). **(C)** Sampling procedure. Each plot was sampled two times, representing the plant developmental stages of flowering (after approx. 60 days) and grain filling (after approx. 90 days). Plots had dimensions of three by four meters and contained four rows of plants. From each plot, three plants, growing near the center of the plot (areas are indicated by a square) were randomly selected and sampled. From each of these plants, three root pieces of secondary order lateral roots of crown roots were sampled. These originated from a depth

10 to 20 cm below the junction of root and shoot. Samples from the three plants grown within one plot (but not from the three experimental blocks) were pooled for further processing. From each sample, bulk soil (BS), rhizosphere collected by manual detachment (rhizosphere stripped, RS), rhizosphere collected by washing (RW), and roots (RT) were collected. **(D)** Sample processing in the laboratory. DNA extraction was performed in random order. Three technical PCR replicates were prepared from each DNA extract. These were pooled per biological sample after PCR and prior to library preparation for sequencing. All samples were randomly assigned to one of two sequencing runs.

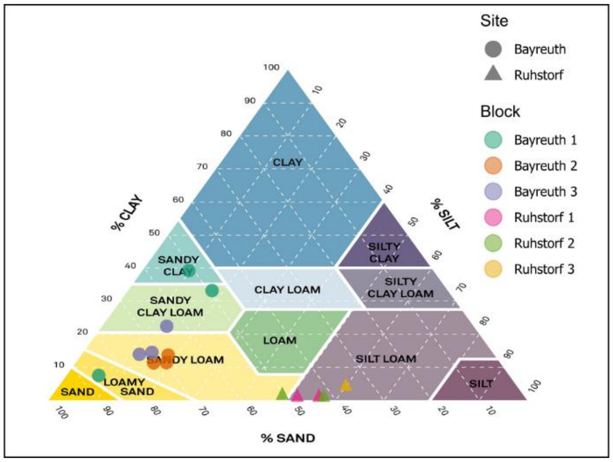

Supplementary Figure 2. Soil texture triangle (METER ENVIRONMENT, Munich, Germany) showing the soil texture at the two field locations. Measurements were made at a depth of 30 cm and within each experimental block. Data and the figure were generated by Tina Köhler and Everton Souza da Silva.

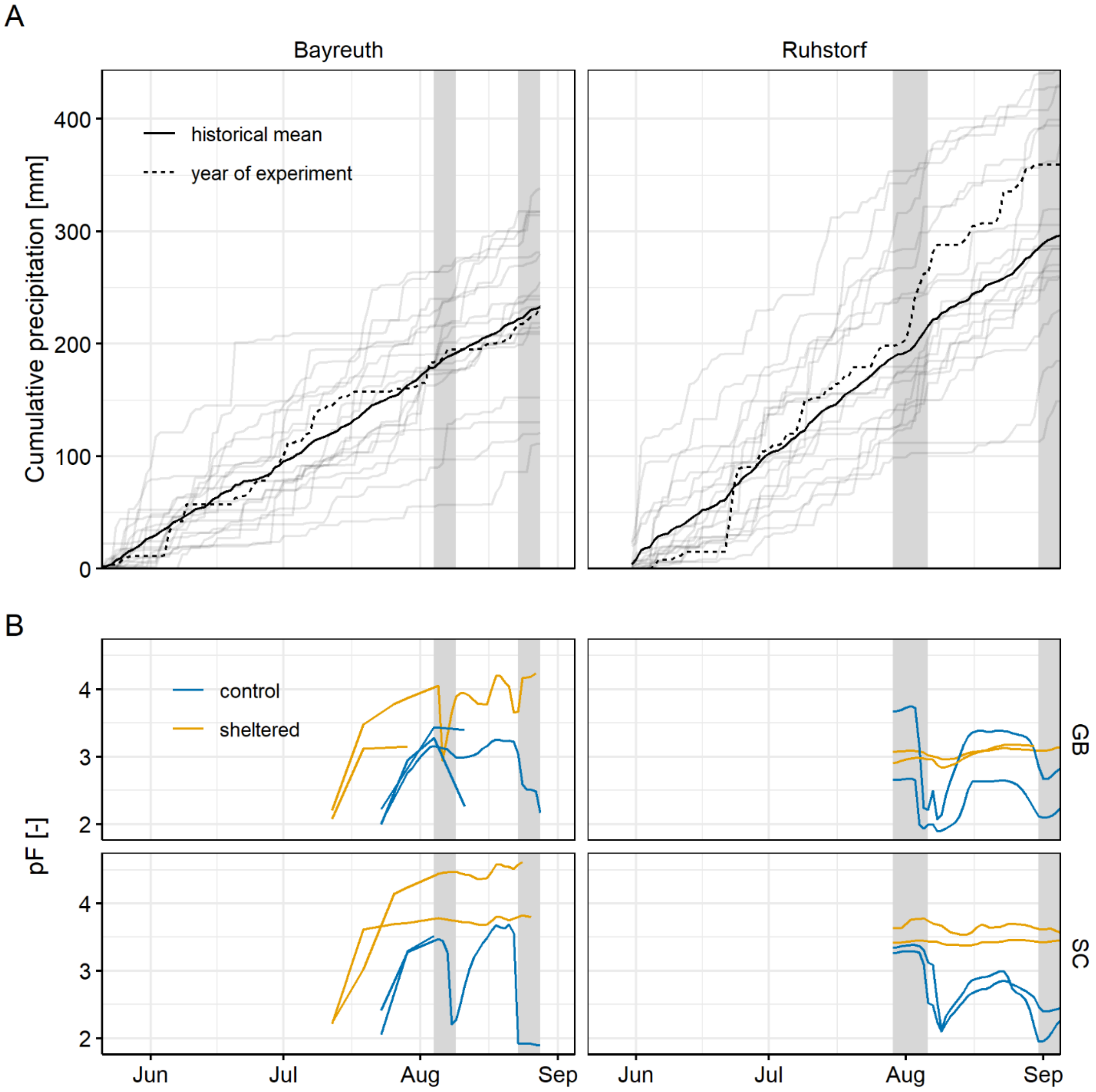

Supplementary Figure 3. Values for cumulative precipitation and soil water potential. Periods when samples were taken are indicated in grey. **(A)** Cumulative precipitation from the day of sowing (Bayreuth: 21/05/2021, Ruhstorf: 31/05/2021) until the start of sampling (Bayreuth: 23/08/2021, Ruhstorf: 31/08/2021). Data was accessed through Meteostat (meteostat.net) from the weather station nearest to the respective field location. For Bayreuth, data was obtained from a weather station in Heinersreuth-Vollhof (lat. 49.96667, long. 11.519692, 4.8 km north-north-west of the field location), and for Ruhstorf, data was obtained from a weather station in Pocking (lat. 48.395245, long. 13.313896, 4.0 km east-north-east of the field location). In addition, we show the cumulative precipitation for the same time range for each year from 2005 to 2023 (no older data is available from the weather station in Pocking) and the mean cumulative precipitation over these years. **(B)** pF of the soil at a depth of 30 cm in a subset of the experimental plots. Interrupted time series are due to equipment failure or occurred when soil water potentials were out of the range that can be measured

with the TEROS21 sensors (METER Environment) used. Data was generated by Tina Köhler. Abbreviations: SC - Braunes Schindelmeiser, GB - Gelber Badischer Landmais

A B C D

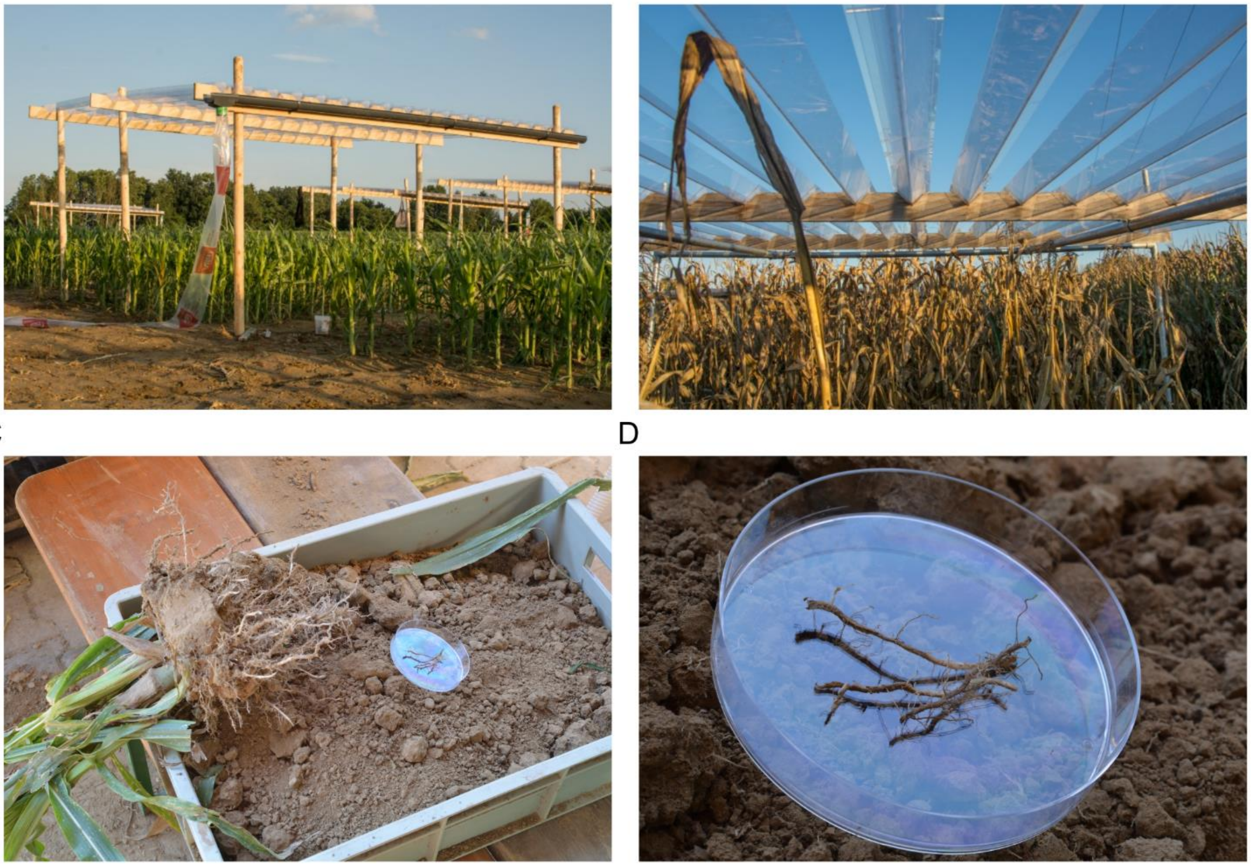

Supplementary Figure 4. **(A)** Rain-out shelter construction. **(B)** Transparent gutters that were used to catch 60% of the precipitation. **(C)** Excavated plant with visible root system. Roots were sampled from 10 to 20 cm below the junction of shoot and roots. **(D)** Exemplary picture of the roots sampled from one plant. Per plot, three such root pieces were sampled from three plants. Material from these nine root pieces was combined for DNA extraction.

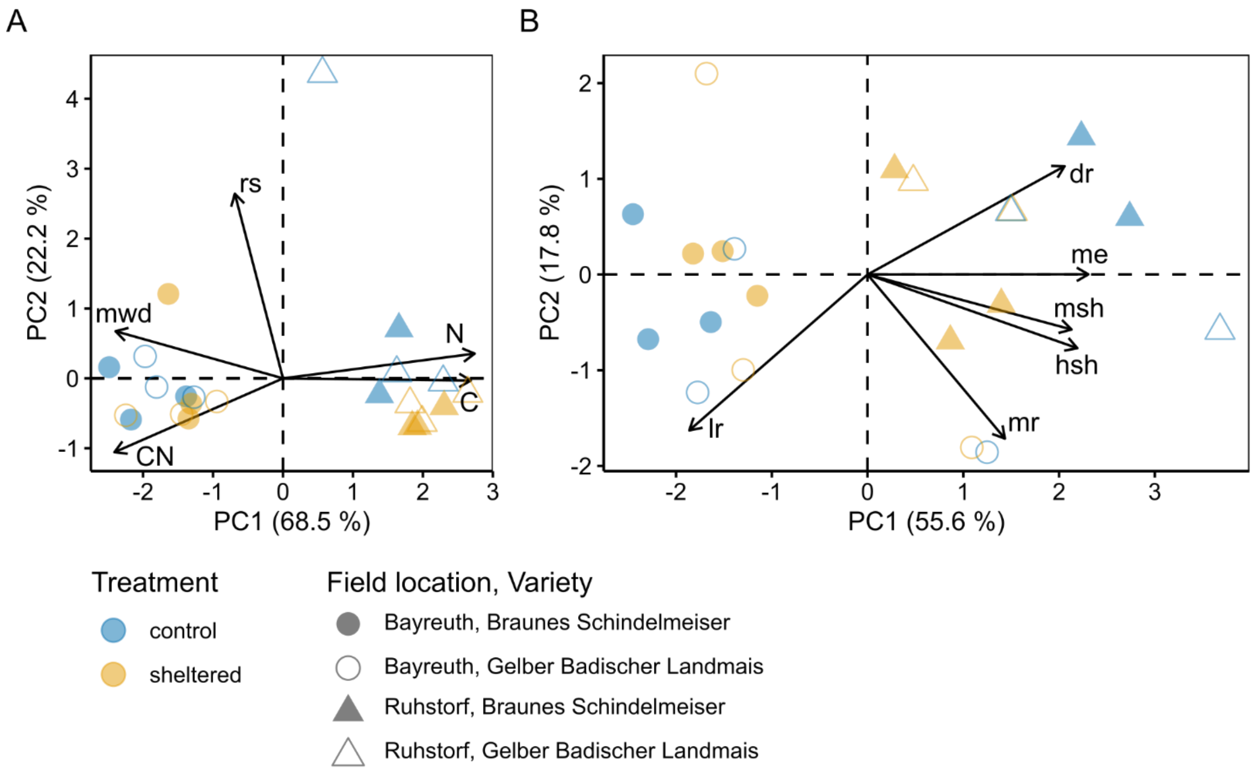

Supplementary Figure 5. Biplots showing results of principal component analyses (PCA). The principal components explaining most of the variance in the datasets are used as axes. Vectors show the direction and magnitude of correlations of the original variables with data points in the 2D space of the two PCA-axes. The proximity of the data points indicates their similarity. PCA were performed after z-transformation of the original variables. All used values are arithmetic means measured on 3 to 4 plants from each experimental plot. **(A)** PCA on rhizosphere soil variables. Abbreviations: mwd – mean weight diameter of soil aggregates determined by wet sieving [µm], N – nitrogen concentration (per unit soil) [mg g^{-1}], C – carbon concentration (per unit soil) [mg g^{-1}], CN – CN-ratio [-], rs – dry mass of rhizosphere soil normalized by the dry biomass of roots [g_{soil} g_{root}^{-1}] Data was generated and provided by Franziska Steiner **(B)** PCA on plant-related variables. Abbreviations: hsh – shoot height measured from the base of the stem to the emergence point of the flag-leaf [cm], msh – total shoot biomass (includes leaf and stem biomass) [g], mr – root biomass [g], lr – total root length [mm], dr – root diameter [mm], me – ear biomass [g]. Data on above-ground variables were generated and provided by Shu-Yin Tung, and data on root parameters by Andreas J. Wild.

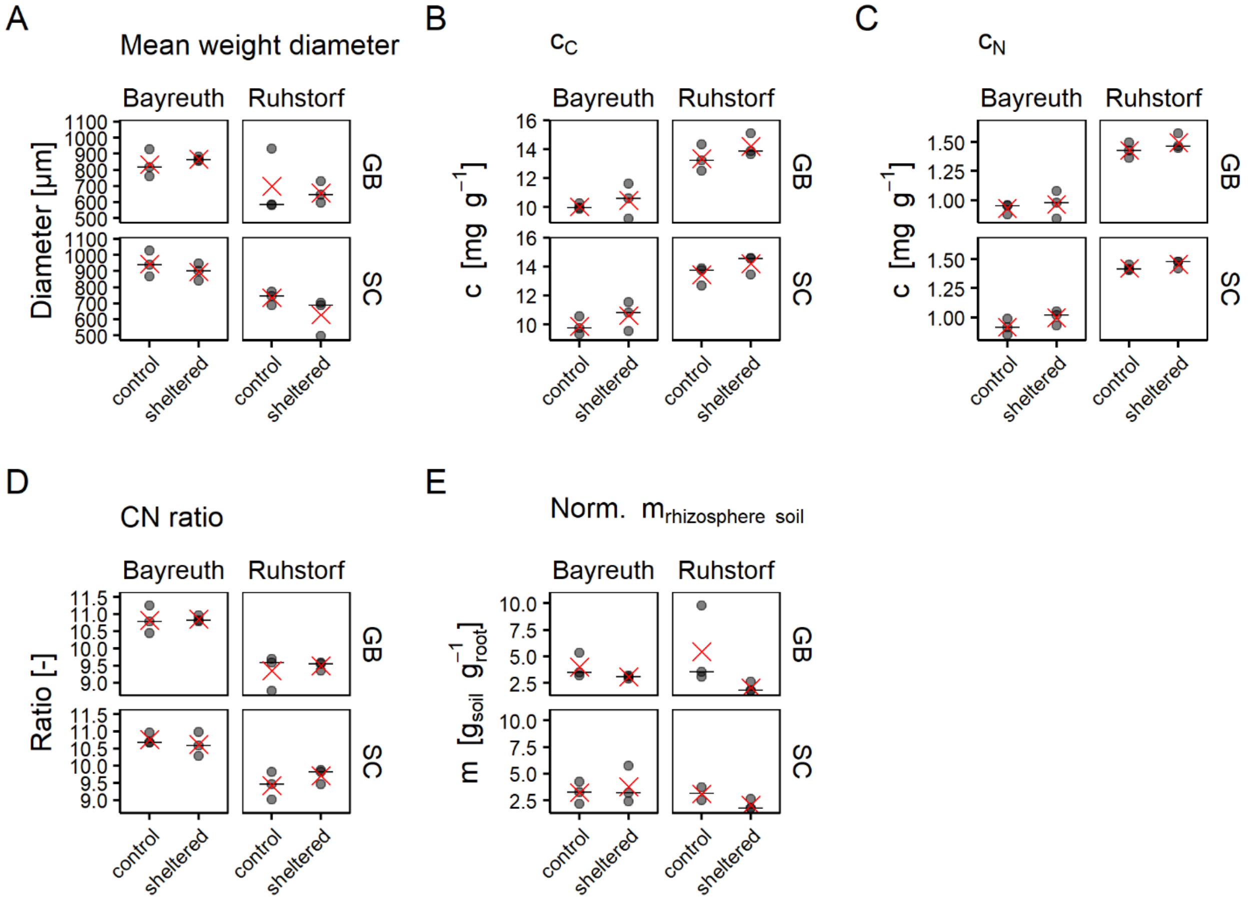

Supplementary Figure 6. Rhizosphere soil-related variables are shown by field location, maize variety, and water availability treatment. All values are arithmetic means measured on 3 to 4 plants from each experimental plot. Medians (black bars) and means (red crosses) per experimental group are shown. **(A)** mean weight diameter of soil aggregates determined by wet sieving, **(B)** carbon concentration per unit soil, **(C)** nitrogen concentration per unit soil, **(D)** CN-ratio, **(E)** dry mass of rhizosphere soil normalized by the dry biomass of roots. Data was generated and provided by Franziska Steiner. Abbreviations: SC: Braunes Schindelmeiser, GB: Gelber Badischer Landmais

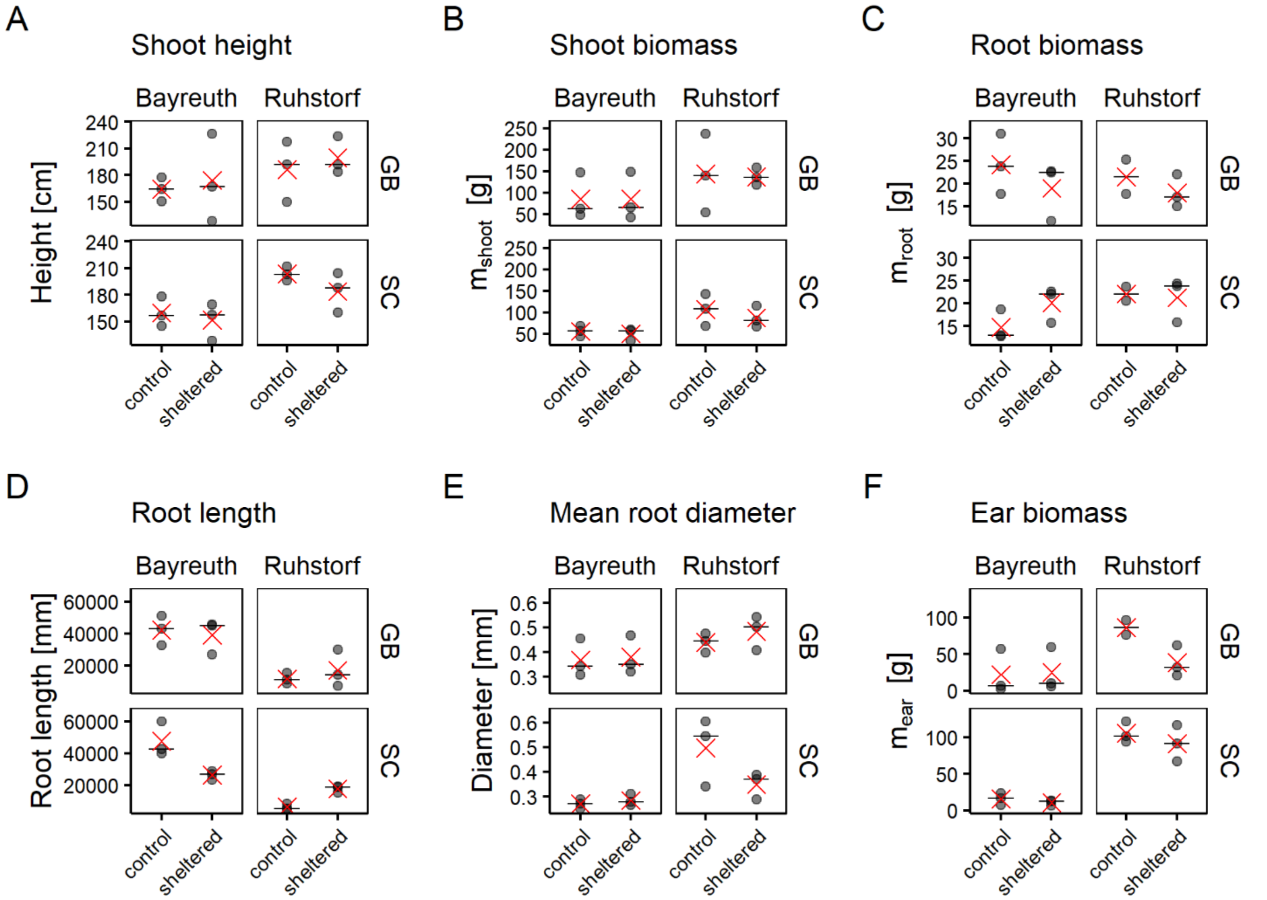

Supplementary Figure 7. Plant-related variables are shown by field location, maize variety, and water availability treatment. Biomass was measured after drying at 105 °C for 24 h. Root variables were obtained from flatbed scanner images analyzed with RhizoVision Explorer (version 2.0.2). All values are arithmetic means measured on 3 to 4 plants from each experimental plot. Medians (black bars) and means (red crosses) per experimental group are shown. **(A)** shoot height measured from the base of the stem to the emergence point of the flag-leaf, **(B)** total shoot biomass (includes leaf and stem biomass), **(C)** root biomass, **(D)** total root length, **(E)** mean root diameter, **(F)** ear biomass. Data on above-ground variables was generated and provided by Shu-Yin Tung, and data on root parameters by Andreas J. Wild. Abbreviations: SC: Braunes Schindelmeiser, GB: Gelber Badischer Landmais

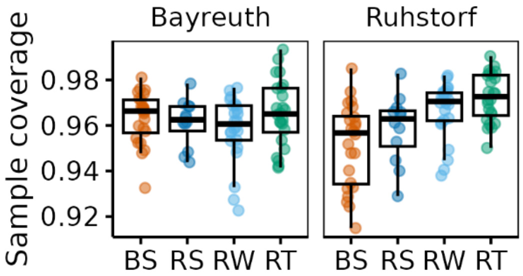

Supplementary Figure 8. Sample coverage (the proportion of the total number of OTUs in the community that belong to the OTUs represented in the sample (Chao and Jost, 2012; Mikryukov and Mahé, 2018)) as calculated with the function phyloseq_coverage from the R library metagMisc (version 0.5.0 (Mikryukov and Mahé, 2018)) shown by field location and compartment.

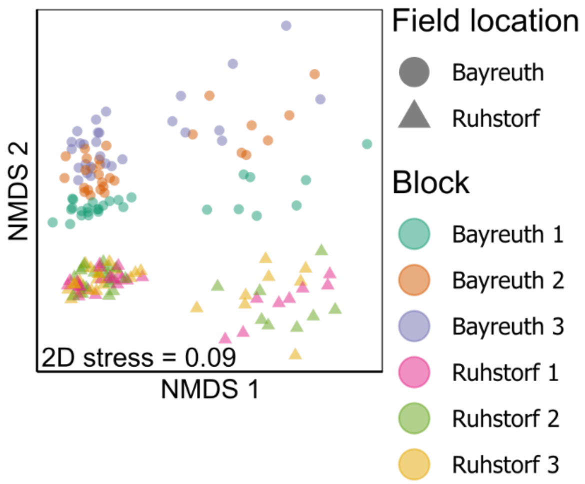

Supplementary Figure 9. Ordination of nMDS on Bray-Curtis similarities between samples calculated on relative abundances of OTUs after rarefaction, square root transformation, and Wisconsin double transformation. Each point represents a sample, and the distance between points reflects the dissimilarity of their communities. Points are colored by experimental blocks.

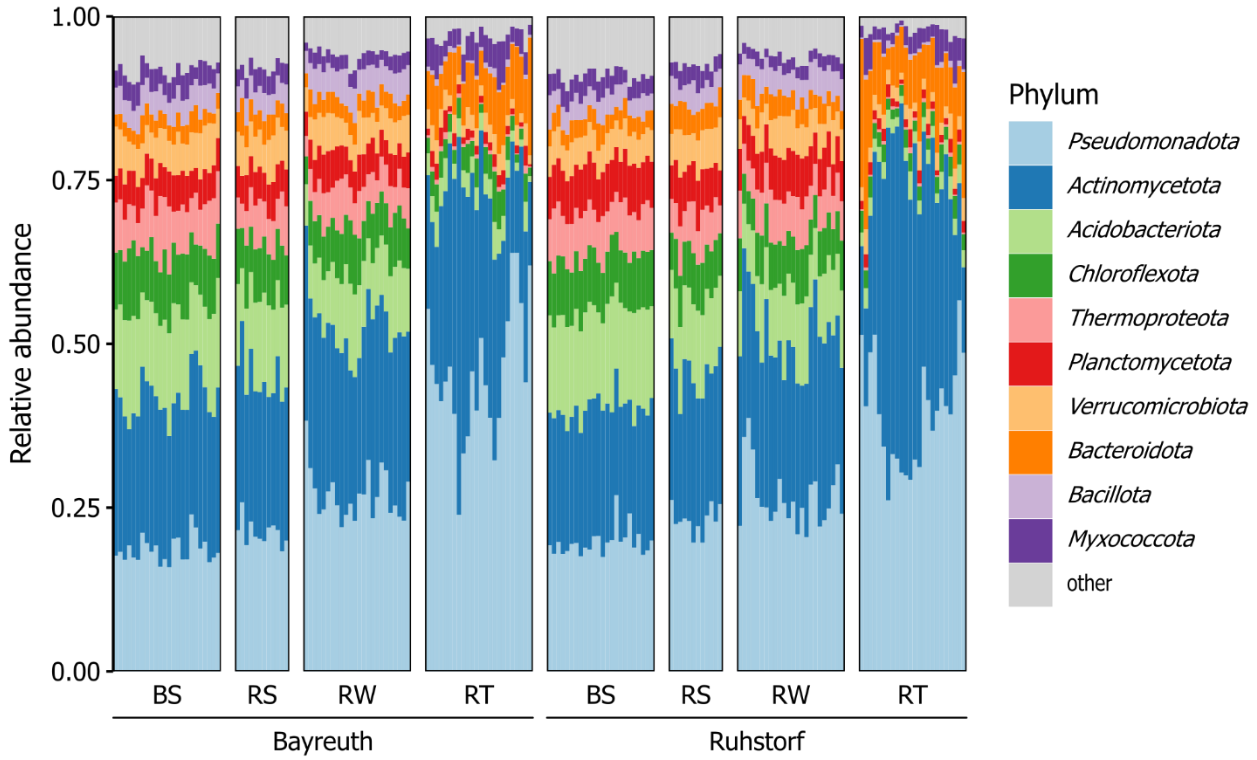

Supplementary Figure 10. Relative abundances are shown at the phylum level for each sample and are grouped by field location and compartment.

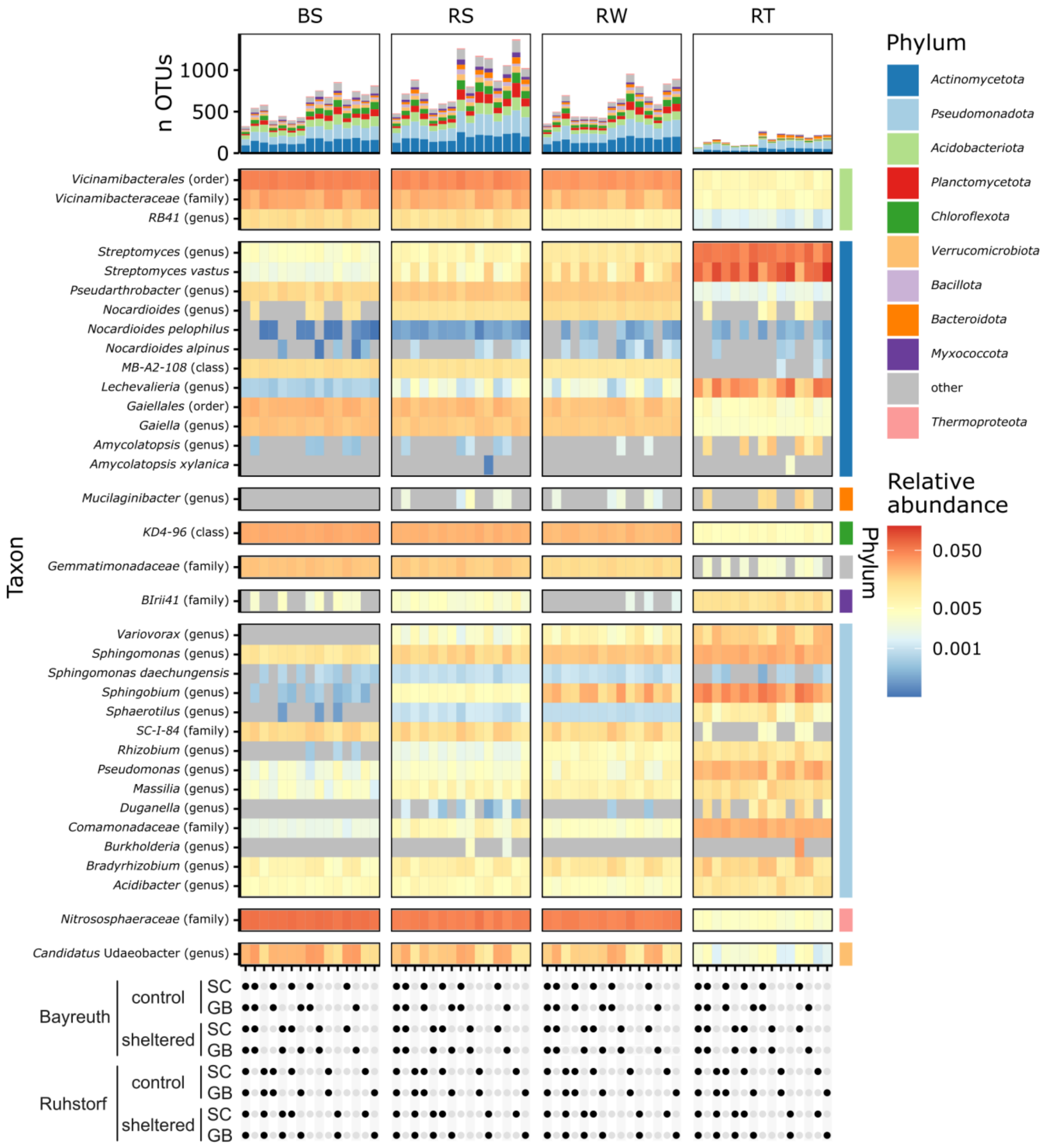

Supplementary Figure 11. Core microbiomes were defined as including those OTUs detected in all samples within a group (occupancy threshold of 100%). Here, the composition of the core microbiome is shown for different groups (subsets of samples). The levels of experimental factors (field location, treatment, and variety) that were included in a group are shown as black dots on the x-axis at the bottom of the figure. The uppermost panel shows the number of core-OTUs at the phylum level for each group. The heatmap shows the average relative abundance at the genus level for those genera with at least a mean relative abundance > 0.015% in one of the groups shown. In the heatmap,

genera are grouped at the phylum level. The values of the color-gradient scale are fractions of 1. Grey tiles indicate that the genus was not a member of the core microbiome for this group of samples.

3.2 Supplementary Tables

Supplementary Table 1. Information on the field locations. Mean annual precipitation was calculated for a period from 2005 to 2022. No older data is available for Ruhstorf. Soils are classified according to the World Reference Base for Soil Resources (2022).

Variable	Bayreuth	Ruhstorf
latitude	49.931907	48.382261
longitude	11.551221	13.263678
elevation	354 m	367 m
mean annual precipitation	685 ± 95 mm	790.5 ± 90 mm
soil classification	Stagnosol	Luvisol
soil organic carbon	10.46 ± 1.07 mg g-1	12.69 ± 0.52 mg g-1
N concentration	0.98 ± 0.09 mg g-1	1.40 ± 0.06 mg g-1
C:N ratio	10.67 ± 0.22	9.09 ± 0.21

Supplementary Table 2. PERMANOVA results on rhizosphere soil variables. PERMANOVA was computed on Euclidean distances calculated on z-transformed data. Due to the low number of possible unique permutations for the factor field location, we consider the *P*-values generated by Monte-Carlo testing (*P*(MC)) more meaningful here. The following variables were included: mean weight diameter of soil aggregates determined by wet sieving, carbon concentration per unit soil, nitrogen concentration per unit soil, CN-ratio, dry mass of rhizosphere soil normalized by the dry biomass of roots. Significant factors (*P*(MC) < 0.05) are shown in bold.

Source	df	SS	MS	Pseudo F	*P* (perm)	Unique perms	*P*(MC)
**field location**	**1**	**75.44**	**75.44**	**37.34**	**0.1034**	**10**	**0.0007**
**treatment**	**1**	**4.91**	**4.91**	**4.29**	**0.0443**	**9968**	**0.0493**
variety	1	0.66	0.66	0.27	0.7576	9712	0.7814
block(field location)	4	8.08	2.02	1.77	0.1487	9946	0.1618
field location × treatment	1	3.33	3.33	2.91	0.1063	9963	0.1001
field location × variety	1	0.64	0.64	0.26	0.7405	9645	0.7766
tretatment × variety	1	1.47	1.47	1.29	0.3096	9962	0.2998
block(field location) × variety	4	9.84	2.46	2.15	0.0889	9947	0.1038

Supplementary Table 3. PERMANOVA results on plant variables. PERMANOVA was computed on Euclidean distances calculated on z-transformed data. Due to the low number of possible unique permutations for the factor field location, we consider the *P*-values generated by Monte-Carlo testing (*P*(MC)) more meaningful here. The following variables were included: shoot height measured from the base of the stem to the emergence point of the flag-leaf, total shoot biomass (includes leaf and stem biomass), root biomass, total root length, mean root diameter, ear biomass. Significant factors (*P*(MC) < 0.05) are shown in bold.

Source	df	SS	MS	Pseudo F	*P* (perm)	Unique perms	*P*(MC)
**field location**	**1**	**48.69**	**48.69**	**10.39**	**0.1055**	**10**	**0.0021**
treatment	1	1.60	1.60	0.32	0.7598	9686	0.8119
variety	1	7.27	7.27	2.46	0.0690	9942	0.0779
block(field location)	4	18.75	4.69	1.59	0.1265	9943	0.1433
field location × treatment	1	3.08	3.08	0.62	0.5790	9647	0.6071
field location × variety	1	6.09	6.09	2.06	0.1076	9952	0.1161
treatment × variety	1	5.71	5.71	1.93	0.1255	9950	0.1336
block(field location) × treatment	4	19.78	4.95	1.67	0.1347	9926	0.1431

Supplementary Table 4. Results of pairwise comparisons using Dunn's tests between compartments within field locations for different α-diversity metrics. Significant (*P* < 0.05) differences are shown in bold.

Metric	Field location	Comparison	*P* (adj.)
Observed	Bayreuth	bulk, rhizosphere (stripped)	0.3220
Observed	Bayreuth	bulk, rhizosphere (washed)	0.1880
**Observed**	**Bayreuth**	**bulk, root**	**< 0.0001**
Observed	Bayreuth	rhizosphere (stripped), rhizosphere (washed)	0.0552
**Observed**	**Bayreuth**	**rhizosphere (stripped), root**	**< 0.0001**
**Observed**	**Bayreuth**	**rhizosphere (washed), root**	**< 0.0001**
Observed	Ruhstorf	bulk, rhizosphere (stripped)	0.2740
**Observed**	**Ruhstorf**	**bulk, rhizosphere (washed)**	**0.0001**
**Observed**	**Ruhstorf**	**bulk, root**	**< 0.0001**
**Observed**	**Ruhstorf**	**rhizosphere (stripped), rhizosphere (washed)**	**0.0298**
**Observed**	**Ruhstorf**	**rhizosphere (stripped), root**	**< 0.0001**
**Observed**	**Ruhstorf**	**rhizosphere (washed), root**	**0.0008**
Shannon	Bayreuth	bulk, rhizosphere (stripped)	0.2810
Shannon	Bayreuth	bulk, rhizosphere (washed)	0.2810
**Shannon**	**Bayreuth**	**bulk, root**	**< 0.0001**
**Shannon**	**Bayreuth**	**rhizosphere (stripped), rhizosphere (washed)**	**0.0435**
**Shannon**	**Bayreuth**	**rhizosphere (stripped), root**	**< 0.0001**
**Shannon**	**Bayreuth**	**rhizosphere (washed), root**	**< 0.0001**
Shannon	Ruhstorf	bulk, rhizosphere (stripped)	0.3740
**Shannon**	**Ruhstorf**	**bulk, rhizosphere (washed)**	**0.0004**
**Shannon**	**Ruhstorf**	**bulk, root**	**< 0.0001**
**Shannon**	**Ruhstorf**	**rhizosphere (stripped), rhizosphere (washed)**	**0.0440**
**Shannon**	**Ruhstorf**	**rhizosphere (stripped), root**	**< 0.0001**
**Shannon**	**Ruhstorf**	**rhizosphere (washed), root**	**0.0004**
Simpson	Bayreuth	bulk, rhizosphere (stripped)	0.2400
Simpson	Bayreuth	bulk, rhizosphere (washed)	0.2760
**Simpson**	**Bayreuth**	**bulk, root**	**< 0.0001**
**Simpson**	**Bayreuth**	**rhizosphere (stripped), rhizosphere (washed)**	**0.0435**
**Simpson**	**Bayreuth**	**rhizosphere (stripped), root**	**< 0.0001**
**Simpson**	**Bayreuth**	**rhizosphere (washed), root**	**< 0.0001**
Simpson	Ruhstorf	bulk, rhizosphere (stripped)	0.9190
**Simpson**	**Ruhstorf**	**bulk, rhizosphere (washed)**	**0.0334**
**Simpson**	**Ruhstorf**	**bulk, root**	**< 0.0001**
Simpson	Ruhstorf	rhizosphere (stripped), rhizosphere (washed)	0.0594
**Simpson**	**Ruhstorf**	**rhizosphere (stripped), root**	**< 0.0001**
**Simpson**	**Ruhstorf**	**rhizosphere (washed), root**	**< 0.0001**

Supplementary Table 5. PERMANOVA results testing the full experimental design on Bray-Curtis-similarities between samples calculated on relative OTU abundance data after square root transformation and Wisconsin double transformation. Significant factors ($P < 0.05$) are shown in bold.

Source	df	SS	MS	Pseudo F	*P* (perm)	Unique perms	$\sqrt{var.}$
**field location**	**1**	**45725.0**	**45725.0**	**9.18**	**0.0043**	**9937**	**22.07**
**compartment**	**3**	**64834.0**	**21611.0**	**10.92**	**0.0001**	**9938**	**21.96**
sampling time	1	2140.2	2140.2	1.17	0.3293	9909	2.25
treatment	1	2828.2	2828.2	1.11	0.3749	9926	1.90
variety	1	2396.2	2396.2	1.09	0.3850	9929	1.58
**block(field location)**	**4**	18349.0	4587.2	2.93	0.0005	9896	10.39
**field location × compartment**	**3**	**14673.0**	**4891.1**	**2.59**	**0.0014**	**9892**	**12.37**
field location × sampling time	1	2044.5	2044.5	1.15	0.3292	9915	2.99
field location × treatment	1	2282.3	2282.3	0.92	0.5343	9942	-2.32
field location × variety	1	2151.3	2151.3	0.98	0.4769	9930	-0.93
compartment × sampling time	2	3788.1	1894.0	1.20	0.2559	9905	3.64
compartment × treatment	3	5568.6	1856.2	1.11	0.3113	9872	3.19
compartment × variety	3	5150.6	1716.9	1.05	0.3973	9860	2.09
sampling time × treatment	1	2144.2	2144.2	1.18	0.3138	9939	3.25
sampling time × variety	1	1943.8	1943.8	1.12	0.3659	9926	2.59
treatment × variety	1	1955.2	1955.2	0.94	0.5112	9939	-1.79
block(field location) × compartment	12	20611.0	1717.6	1.10	0.2248	9773	4.78
block(field location) × sampling time	4	6789.9	1697.5	1.09	0.3401	9856	3.41
**block(field location) × treatment**	**4**	**9969.3**	**2492.3**	**1.60**	**0.0212**	**9889**	**8.16**
block(field location) × variety	4	8675.3	2168.8	1.39	0.0671	9889	6.61
field location × compartment × sampling time	2	3354.1	1677.1	1.06	0.4065	9894	2.90
field location × compartment × treatment	3	4965.3	1655.1	1.01	0.4653	9859	1.31
field location × compartment × variety	3	5191.4	1730.5	1.06	0.3876	9890	3.15
field location × sampling time × treatment	1	1843.6	1843.6	1.03	0.4315	9950	1.92
field location × sampling time × variety	1	1980.9	1980.9	1.13	0.3433	9930	3.94
field location × treatment × variety	1	1691.0	1691.0	0.82	0.6174	9927	-4.39
compartment × sampling time × treatment	2	3619.2	1809.6	1.12	0.3428	9919	4.06
compartment × sampling time × variety	2	3331.0	1665.5	1.07	0.4036	9913	2.96
compartment × treatment × variety	3	5040.8	1680.3	1.04	0.4072	9874	2.79
sampling time × treatment × variety	1	1735.8	1735.8	1.02	0.4413	9926	1.62
block(field location) × compartment × sampling time	8	12611.0	1576.4	1.02	0.4564	9834	2.48
block(field location) × compartment × treatment	12	19286.0	1607.1	1.03	0.4193	9752	3.50
block(field location) × compartment × variety	12	19246.0	1603.9	1.03	0.4212	9779	3.64
block(field location) × sampling time × treatment	4	6854.5	1713.6	1.09	0.3320	9863	4.95
block(field location) × sampling time × variety	4	6705.6	1676.4	1.08	0.3566	9881	4.52
block(field location) × treatment × variety	4	8329.1	2082.3	1.34	0.1042	9885	8.66
field location × compartment × sampling time × treatment	2	3733.1	1866.5	1.16	0.3098	9915	6.51
field location × compartment × sampling time × variety	2	3325.5	1662.7	1.07	0.4015	9922	4.14
field location × compartment × treatment × variety	3	5442.8	1814.3	1.12	0.2974	9884	6.55
field location × sampling time × treatment × variety	1	1701.1	1701.1	1.01	0.4517	9936	1.67
compartment × sampling time × treatment × variety	2	3178.3	1589.2	1.02	0.4509	9935	2.50
block(field location) × compartment × sampling time × treatment	8	12897.0	1612.1	1.04	0.4200	9816	5.49
block(field location) × compartment × sampling time × variety	8	12480.0	1560.0	1.01	0.4863	9836	2.03
block(field location) × compartment × treatment × variety	12	19126.0	1593.8	1.02	0.4338	9772	4.58
block(field location) × sampling time × treatment × variety	4	6715.8	1678.9	1.08	0.3619	9888	6.51
field location × compartment × sampling time × treatment × variety	2	3080.5	1540.3	0.99	0.4838	9924	-1.96
residual	8	12414.0	1551.8				39.39

Supplementary Table 6. Results of pairwise PERMANOVA on Bray-Curtis-similarities comparing levels of the factor field location within all levels of the factor “compartment”. Due to the low number of possible unique permutations, we consider the *P*-values generated by Monte-Carlo testing (*P*(MC)) more meaningful here. Significant (*P*(MC) < 0.05, Monte-Carlo *P*-values) differences between levels are shown in bold.

Compartment	Comparison	t	*P*(perm)	Unique perms	*P*(MC)
**bulk**	**Bayreuth, Ruhstorf**	**2.6939**	**0.1032**	**10**	**0.0001**
**rhizosphere (stripped)**	**Bayreuth, Ruhstorf**	**2.1526**	**0.0982**	**10**	**0.0003**
**rhizosphere (washed)**	**Bayreuth, Ruhstorf**	**2.6188**	**0.1045**	**10**	**0.0001**
**root**	**Bayreuth, Ruhstorf**	**2.4015**	**0.1022**	**10**	**0.0001**

Supplementary Table 7. Results of pairwise PERMANOVA on Bray-Curtis-similarities comparing levels of the factor “compartment” within both levels of the factor field location. Significant (*P* < 0.05) differences between levels are shown in bold.

Field location	Comparison	t	*P*(perm)	Unique perms
**Bayreuth**	**rhizosphere (washed), bulk**	**1.4963**	**0.0001**	**9732**
**Bayreuth**	**rhizosphere (washed), rhizosphere (stripped)**	**1.2869**	**0.0001**	**9719**
**Bayreuth**	**rhizosphere (washed), root**	**3.1542**	**0.0001**	**9819**
Bayreuth	bulk, rhizosphere (stripped)	1.0638	0.0971	9728
**Bayreuth**	**bulk, root**	**3.5159**	**0.0001**	**9825**
**Bayreuth**	**rhizosphere (stripped), root**	**2.7212**	**0.0001**	**9853**
**Ruhstorf**	**rhizosphere (washed), bulk**	**2.0392**	**0.0001**	**9755**
**Ruhstorf**	**rhizosphere (washed), rhizosphere (stripped)**	**1.332**	**0.0001**	**9742**
**Ruhstorf**	**rhizosphere (washed), root**	**3.5664**	**0.0001**	**9832**
**Ruhstorf**	**bulk, rhizosphere (stripped)**	**1.3339**	**0.0001**	**9644**
**Ruhstorf**	**bulk, root**	**4.183**	**0.0001**	**9863**
**Ruhstorf**	**rhizosphere (stripped), root**	**2.9755**	**0.0001**	**9844**

Supplementary Table 8. Results of pairwise comparisons of dispersion with PERMDISP on Bray-Curtis-similarities. Significant (*P* < 0.05) differences between levels are shown in bold.

Factor	Level	Comparison	t	*P*(perm)
**compartment**	**bulk**	**Bayreuth, Ruhstorf**	**57.535**	**0.0001**
compartment	rhizosphere (stripped)	Bayreuth, Ruhstorf	19.163	0.0704
**compartment**	**rhizosphere (washed)**	**Bayreuth, Ruhstorf**	**36.428**	**0.0006**
compartment	root	Bayreuth, Ruhstorf	15.539	0.1358
field location	Bayreuth	bulk, rhizosphere (stripped)	18.111	0.2477
field location	Bayreuth	bulk, rhizosphere (washed)	0.32357	0.7474
**field location**	**Bayreuth**	**bulk, root**	**66.895**	**0.0001**
field location	Bayreuth	rhizosphere (stripped), rhizosphere (washed)	14.082	0.3727
**field location**	**Bayreuth**	**rhizosphere (stripped), root**	**59.967**	**0.0001**
**field location**	**Bayreuth**	**rhizosphere (washed), root**	**67.146**	**0.0001**
field location	Ruhstorf	bulk, rhizosphere (stripped)	0.5953	0.9251
field location	Ruhstorf	bulk, rhizosphere (washed)	19.982	0.0628
**field location**	**Ruhstorf**	**bulk, root**	**15.61**	**0.0001**
field location	Ruhstorf	rhizosphere (stripped), rhizosphere (washed)	12.856	0.8133
**field location**	**Ruhstorf**	**rhizosphere (stripped), root**	**12.341**	**0.0001**
**field location**	**Ruhstorf**	**rhizosphere (washed), root**	**14.616**	**0.0001**

4 Literature cited in the Supplementary Material

Chao, A., and Jost, L. (2012). Coverage-based rarefaction and extrapolation: standardizing samples by completeness rather than size. *Ecology* 93, 2533–2547. doi: 10.1890/11-1952.1

IUSS Working Group WRB (2022). *World Reference Base for Soil Resources. International soil classification system for naming soils and creating legends for soil maps. 4th edition.* Vienna, Austria: International Union of Soil Sciences (IUSS).

Mikryukov, V., and Mahé, F. (2018). metagMisc: Miscellaneous functions for metagenomic analysis. Available at: https://github.com/vmikk/metagMisc (Accessed September 25, 2023).
